# ADAM11 a novel regulator of Wnt and BMP4 signaling in neural crest and cancer

**DOI:** 10.3389/fcell.2023.1271178

**Published:** 2023-09-12

**Authors:** Ankit Pandey, Hélène Cousin, Brett Horr, Dominique Alfandari

**Affiliations:** Department of Veterinary and Animal Sciences, University of Massachusetts Amherst, Amherst, MA, United States

**Keywords:** neural crest, ADAM, BMP4, Wnt, development, cancer

## Abstract

**Introduction:** Cranial neural crest (CNC) cells are induced at the border of the neural plate by a combination of FGF, Wnt, and BMP4 signaling. CNC then migrate ventrally and invade ventral structures where they contribute to craniofacial development.

**Methods:** We used loss and gain of function experiments to determine phenotypes associated with the perturbation of Adam11 expression in *Xenopus Laevis*. Mass spectrometry to identify partners of Adam11 and changes in protein expression in CNC lacking Adam11. We used mouse B16 melanoma to test the function of Adam11 in cancer cells, and published database analysis to study the expression of ADAM11 in human tumors.

**Results:** Here we show that a non-proteolytic ADAM, Adam11, originally identified as a putative tumor suppressor binds to proteins of the Wnt and BMP4 signaling pathway. Mechanistic studies concerning these non-proteolytic ADAM lack almost entirely. We show that Adam11 positively regulates BMP4 signaling while negatively regulating β-catenin activity. *In vivo*, we show that Adam11 influences the timing of neural tube closure and the proliferation and migration of CNC. Using both human tumor data and mouse B16 melanoma cells, we further show that ADAM11 levels similarly correlate with Wnt or BMP4 activation levels.

**Discussion:** We propose that ADAM11 preserves naïve cells by maintaining low Sox3 and Snail/Slug levels through stimulation of BMP4 and repression of Wnt signaling, while loss of ADAM11 results in increased Wnt signaling, increased proliferation and early epithelium to mesenchyme transition.

## Introduction

The development of neural crest cells during embryogenesis involves both pathways that regulate cellular migration and invasion, which are comparable to the most aggressive tumors (Medina-Cuadra and Monsoro-Burq, 2021). Often, tumorigenesis involves a first step in which cells lose their fully differentiated state to revert to a more naïve pluripotent state. In addition, once the original tumor is formed, the cells may undergo epithelium to mesenchyme transition to evade the original tissue and invade a new target site where they create metastasis (Chaffer et al., 2016; Wilson et al., 2020). Understanding the molecular underpinnings of this transition is critical for developing strategies to stop it in tumors (Shibue and Weinberg, 2017).

Here, we investigated the role of non-proteolytic ADAM proteins in EMT and cellular migration in the cranial neural crest cells. In the embryo, the cranial neural crest cells are induced at the border of the neural and non-neural ectoderm by signals emanating from the surrounding tissues that include Wnt, FGF, and BMP4 (Bastidas et al., 2004; Steventon et al., 2009; de Croze et al., 2011; Garnett et al., 2012). Once induced, the CNC cells remain stem-like and progress through an EMT to migrate and invade the ventral structures of the embryo, where they differentiate into bone cartilage and ganglia of the face (Alfandari et al., 2010; Scarpa et al., 2015).

While other ADAMs have a well-established role in CNC migration (Alfandari et al., 2001; McCusker et al., 2009; Neuner et al., 2009; Cousin et al., 2012; Khedgikar et al., 2017), the role of non-proteolytic ADAMs, also expressed in this tissue, during development has not been studied. These ADAMs are single-pass transmembrane proteins that possess the same domain organization as other members of the family, including a pro-domain, metalloprotease domain, disintegrin and cysteine-rich domain, EGF domain, and a short transmembrane and cytoplasmic domain. What makes them stand out is that the key glutamic acid in the protease active site is not present, rendering them proteolytically dead (Hsia et al., 2019). Proteolytic ADAMs control Notch, EGF, TGF, Ephrin, and Wnt signaling pathways by cleaving substrate or receptors to activate or inhibit the relevant signaling pathways (Alfandari et al., 2009; Reiss and Saftig, 2009; Klein and Bischoff, 2011). In contrast, non-proteolytic ADAMs have been shown to interact with multiple transmembrane proteins, including integrins, LGI, and Kv channels, and to play a significant role in synapse organization that establishes sensory neural circuits (Sagane et al., 2008; Wang et al., 2018; Hsia et al., 2019).

We have previously shown that *Adam11* is expressed in the cranial neural crest and a subset of neurons in the early neural tube in *Xenopus laevis* (Cai et al., 1998). A very similar expression pattern was described in the mouse embryo (Rybnikova et al., 2002; Diez-Roux et al., 2011). Mice lacking *Adam11* are viable yet have deficiencies in pain perception, suggesting a key role for the protein in establishing the normal neuronal connections responsible (Takahashi et al., 2006a; Takahashi et al., 2006b). Here, we show that loss of Adam11 during early embryogenesis results in early neural tube closure and early EMT of the CNC *in vitro*. We further show that Adam11 promotes BMP4 signaling and reduces Wnt/β-catenin signaling *in vivo*. The Wnt signaling pathway has been directly involved in the increased proliferation through control of *CyclinD1* (Koehler et al., 2013; Kafri et al., 2016) and EMT, with the increase in *Snail* transcription factors expression driving the loss of epithelial cadherins and the progression of EMT (Yook et al., 2005). Loss of Adam11 in embryos results in an increased β-catenin activation, increased expression of *Snai2* and *CyclinD1*, and increased CNC proliferation.

Interestingly, a decrease in *ADAM11* expression is found in the majority of solid tumors that have been studied and correlates with an increase in Wnt signaling and *CYCLIND1* expression (this study). Decreasing Adam11 in B16 mouse melanoma cells similarly increases cell proliferation by promoting the G1-S transition, a step controlled by CyclinD1. We also show that Adam11 increases BMP4 signaling resulting in increased Smad1-5 phosphorylation and a decrease in Sox3 expression, suggesting that Adam11 reduces Sox3 expression in the naïve CNC to prevent their early differentiation. Similarly, in the neural crest derived tumor, neuroblastoma, *ADAM11* expression is higher than in the normal tissue, with a concurrent increase in *Smad1-5* expression and decreases in *Sox3* expression.

We propose that Adam11 maintains stem cell-like attributes in both CNC and cancer and that either an increase in Adam11 or a decrease in Adam11 can create an imbalance toward stemness or EMT, contributing to the cancer pathogenicity.

## Results

### Adam11 knockdown (KD) accelerates neural tube closure

**FIGURE 1:**
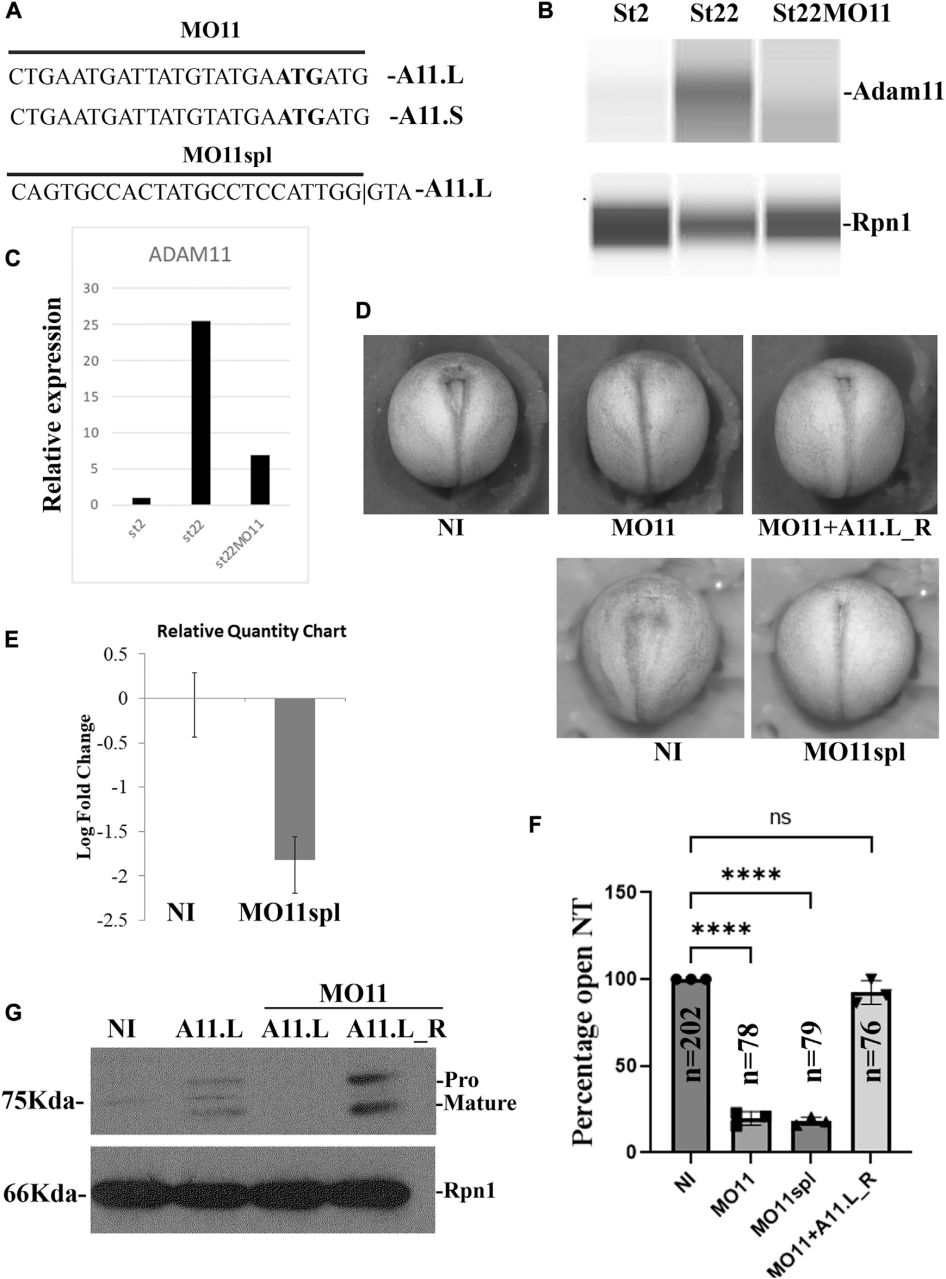
Adam11 Knock Down. **(A)** Morpholino design. Coding sequences for *Adam11.L* and S with the translation start (ATG) bolded matching the translation-blocking morpholino (MO11). **(B)** Capillary Western blot with a monoclonal antibody (mAb) to Adam11 (DA3C5) using membrane extract from 10 embryos at stage 2 and 22. Knockdown efficiency of a translation-blocking MO for Adam11 (MO11) was tested on either non-injected control embryos or embryos injected at the one-cell stage with 12.5 ng of MO11. **(C)** Quantitative representation of the capillary western bot from **(B)** using ribophorin 1 (Rpn1) to normalize. **(D)** Representative photograph of neurula stage embryos (Dorsal view). Anterior is up. Embryos were injected at the one-cell stage with either the translational blocking MO11 or Splicing blocking MO11spl. Embryos injected with MO11 were rescued using 12.5 pg of ADAM11_R rescue mRNA, lacking the morpholino binding sequence. **(E)** The histogram represents RT-qPCR from embryos injected with Adam11.L splice-blocking morpholino. The oligonucleotides are designed to only amplify the spliced mRNA. **(F)** Histogram representing statistical analysis of the phenotype represented in **(D)**. Each dot represents one biological replicate, the total number of embryos analyzed for each condition is given (n). **(G)** Western blot of Adam11.L-flag. *Adam11.L* mRNA (1 ng) was injected at the one-cell stage either alone or with MO11 (12.5 ng). Adam11.L_R rescue lacking the 5′untranslated sequence is translated even in the presence of MO11. Asterisks represent the statistical significance (ANOVA) at *p* < 0.0001 (****).

*Adam11*.*L* is expressed in the central nervous system and the cranial neural crest (Cai et al., 1998). The mRNA for both S and L allo-alleles is expressed zygotically at low levels. *Adam11*.*S* is the major form expressed during gastrulation, while *Adam11*.*L* is the main form during neural tube closure and neural crest cell migration (Xenbase RRID: SCR_003280, st17 to 25) (Session et al., 2016; Fortriede et al., 2020). To test the role of Adam11, we designed two sets of morpholino to either block translation of both the *Adam11*.*L* and.*S* allo-alleles (MO11, L and S) or block the splicing of the first intron of *Adam11.L* which is the main form expressed within the cranial neural crest cells (MO11spl) (Figure 1A). The efficiency of these morpholinos was tested by quantitative capillary Western blot (Figures 1B, C) and RT-qPCR (Figure 1E), we also confirmed that MO11 blocks translation of 1 ng of mRNA encoding for *Adam11*.*L* (Figure 1G, lane2, A11.L) but not the translation of an mRNA lacking the 5′UTR corresponding to the morpholino binding site (Figure 1G, lane4, A11.L_R). We used time-lapse imaging to follow potential developmental defects after the onset of zygotic transcription (Stage 8). While we did not see any defect or delay during gastrulation, we observed that the neural tube closed earlier in morphant embryos compared to control embryos (Figures 1D, F). This observation was true for both splicing and translation-blocking morpholino and is a very rare phenotype as injections typically result in a slight delay of development but rarely an apparent acceleration (DA personal observation). We were able to rescue this phenotype by injecting Adam11.L mRNA lacking the 5′ untranslated region (Adam11.L_R), a form that is not affected by the morpholino (Figures 1D, F), confirming that the phenotype was indeed specific (Figures 1F, G). Given that both Morpholinos gave the same phenotypes, we focused the rest of the study on the translation-blocking morpholino that blocked both allo-alleles.

### Loss of Adam11 affects neural crest cell migration

**FIGURE 2:**
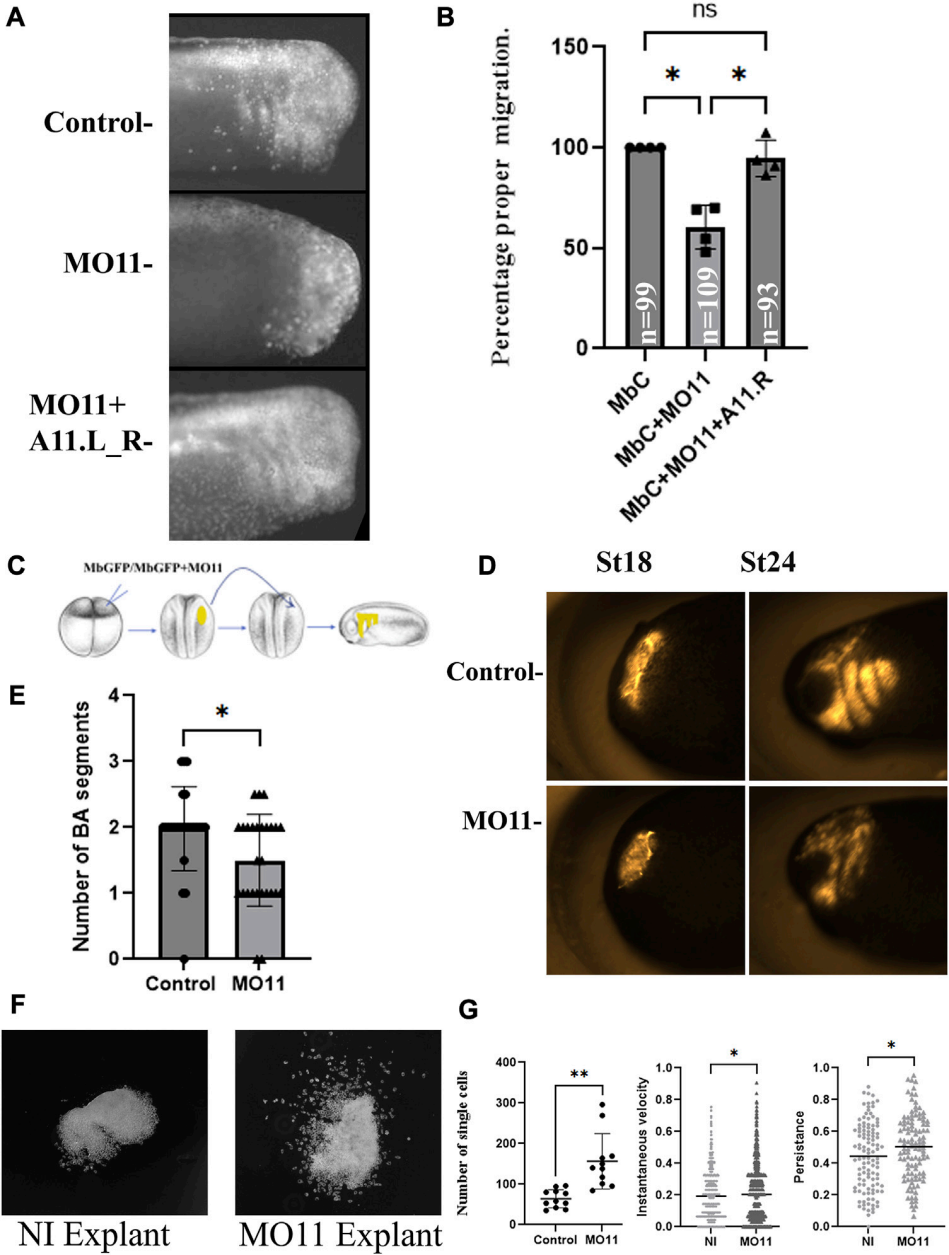
Adam11 Knock down affects cranial neural crest cell migration. **(A)** Fluorescent tracking of CNC migration. Lineage tracer (MbC; Membrane Cherry) was injected at the 8-cell-stage in one dorsal animal blastomere, either alone (control) or with the morpholino to Adam11 (1.56 ng) or both MO11 and the Adam11L_R rescue mRNA (125 pg). The presence or absence of fluorescent neural crest on the lateral and ventral side of the embryo is scored only in embryos with proper targeting (Dorsal anterior quadrant). **(B)** Statistical analysis of the targeted injection. The percentage of embryos with fluorescent CNC in the migration pathway is represented (*Y*-axis) normalized to embryos injected with Membrane Cherry alone (100%). Individual dots, squares and triangles represent each biological replicates (4). Asterisks represent the *p*-value for ANOVA< 0.05. **(C)** Schematic diagram of CNC grafting experiment. **(D)**. Representative photographs of embryo grafted with fluorescent CNC explant at stage 18 and scored at stage 24 for both control and MO11 injected embryos. **(E)** Statistical analysis of the grafting experiment (n is the number of grafted embryos). The histogram represents the number of branchial arches (BA) segments scored at stage 24. Each dot or triangle represents one embryo from five biological replicates (** = *p* < 0.01). **(F)** Representative example of Cranial neural crest cell explants on fibronectin. Photographs were taken during the initial phase of collective cell migration (5 h). **(G)** Statistical analysis of cellular migration showing the number of single cells per explant, the instantaneous velocity and the persistence of cell migration. Single cells were scored at 5 h. Each dot represents one explant. The instantaneous velocity was measured for 110 single cells over (30 min). The persistence of migration was measured on the same cells by dividing the distance from the start and end position by the distance traveled. At least 3 biological replicates for each experiment. Student’s t-test was applied (**=*p* < 0.01, *=*p* < 0.05).

Because of *Adam11*.*L* expression in the cranial neural crest cells (CNC), we tested the ability of CNC with reduced Adam11 protein to migrate *in vivo* and *in vitro* (Figure 2). *In vivo,* we tested the migration capacity by targeted injection (Figures 2A, B) as well as grafting experiments (Figures 2D, E) using previously described assays (Cousin et al., 2011; Cousin et al., 2012; Abbruzzese et al., 2016). In both experiments, we found that the loss of Adam11 perturbed CNC migration significantly in approximately 50% of the embryos (Figures 2B, E). In the grafting experiments, we found significant defects in the CNC migration into the Branchial segments but not in the mandibular pathway. The inhibition of CNC migration was rescued by injection of the Adam11.L rescue mRNA in the CNC precursor cells (Figures 2A, B). As we have shown before, inhibition of CNC migration *in vivo* can be due to either a loss of cell motility (Alfandari et al., 2003) or a more complex effect of the cellular environment (3D ECM with multiple structural and signaling proteins) *in vivo* (Cousin et al., 2012). To test this possibility, we placed CNC explants *in vitro* on fibronectin-coated substrates and performed time-lapse video microscopy. Using this assay, we have previously shown that CNC explant dissected at stage 17 migrates collectively during the first 5 h before individual cells break away, a process also observed *in vivo* (Alfandari et al., 2001; Alfandari et al., 2003). We found that CNC cells in both control and Adam11 KD were able to spread and migrate on the substrate efficiently, but that cells lacking Adam11 migrated individually even during the first 5 h (Figure 2F). Furthermore, we found that the average velocity and persistence of migration was higher in cells with reduced Adam11 (Figure 2G). Collectively, these results show that Adam11 is essential for proper CNC migration and may influence the timing of neural tube closure and single neural crest cell migration. We then tested if neural and neural crest cell markers were expressed and localized properly in embryos lacking Adam11.

### Loss of Adam11 affects neural and neural crest cell markers at the neurula stage

**FIGURE 3:**
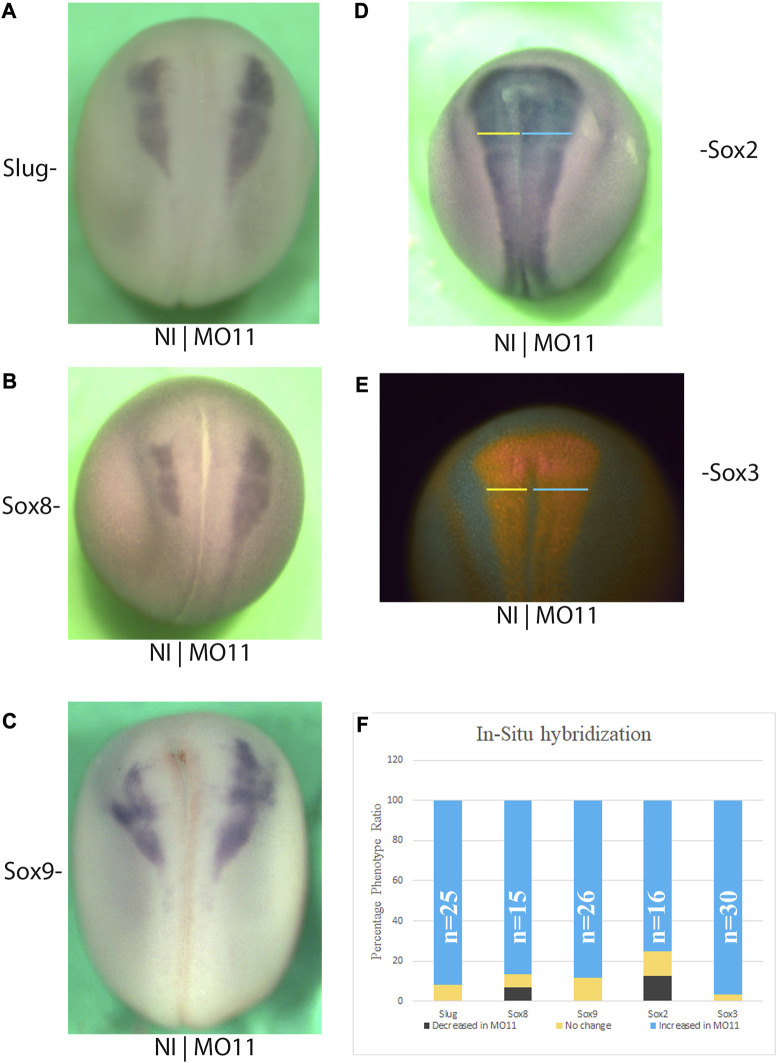
Adam11 KD affects neural and neural crest cell markers at neurula stage. Dorsal views of representative *in situ* Hybridization with neural crest **(A–C)** and Neural markers **(D)**. The injected side is to the right, and the anterior is toward the top. **(E)** Immunodetection of the neural marker Sox3 with the same orientation as above. Colored lines in **(D,E)** highlight the increased size of the neural plate on the injected side. **(F)** Histogram depicting phenotype of the above-mentioned markers. Black represents a decrease, yellow no change and, cyan an increase of the marker in the injected side compared to the control side. The number of embryos analyzed in each condition is indicated (n).

Given the distribution of Adam11.L mRNA in the CNC and neural tube, and the phenotypes observed following Adam11 KD, we focused our analysis on neural and neural crest markers at stages when the first phenotype is visible (Neurula stage). We used the well-characterized neural crest marker *Slug/Snai2* (Figure 3A), *Sox8* (Figure 3B), and *Sox9* (Figure 3C) in embryos injected unilaterally at the 2-cell stage with MO11 and a fluorescent lineage tracer. This allows KD in one-half of the embryo (right or left side). *In-situ* hybridization revealed that all three markers were expressed at the appropriate position. Interestingly, we found that these markers were increased on the injected side (Right and Figure 3F). Expansion of the neural crest territory can, in some cases, be done at the expense of the neural ectoderm. We tested the position of two neural markers, *Sox2* (Figure 3D) by *in situ* hybridization and Sox3 by immunostaining (Figure 3E). In both cases, we found that the domain of expression on the injected side was also wider, suggesting that the increase in neural crest territory was not done at the expense of the neural tissue.

### Loss of Adam11 increases β-catenin activity in the CNC

**FIGURE 4:**
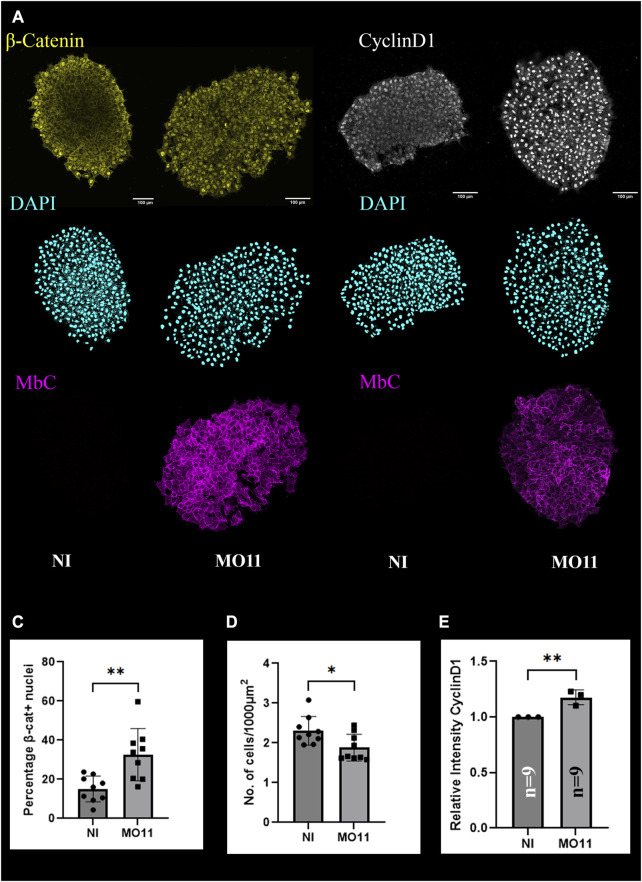
Adam11 KD increases β-catenin activity in the CNC. **(A,B)** confocal imaging of CNC explants 1 hour after dissection. CNC explants were dissected at stage 17 and placed on fibronectin-coated glass coverslip. Explants were stained for β-catenin (A, yellow), Cyclin-D1 (B, white), and nuclei (DAPI, cyan). Membrane cherry (MbC, magenta) was injected with MO11 to identify the cells with KD of Adam11. **(C)** Histogram representing the percentage of β-catenin positive nuclei. **(D)** Histogram representing the number of nuclei per area. **(E)** Histogram depicting the overall relative intensity of CyclinD1 immunofluorescence between control CNC explants and Adam11 knockdown explants. Student’s t-test was done for statistical analysis (*=*p* < 0.05, **=*p* < 0.01).

Given the early onset of single CNC cell migration and increased *Slug* expression, we hypothesized that β-catenin controlled EMT could be dysregulated following Adam11 KD. To test this hypothesis, we dissected CNC explants from embryos injected into one cell of the two-cell stage and compared the explants from the control and the injected side after migration on fibronectin-coated slides (Figure 4). Membrane Cherry was co-injected with the morpholino to identify the injected side and confirm that the CNC had been targeted. We found that while the overall fluorescence of β-catenin was similar on the control and injected side, the percentage of nuclei with β-catenin protein was expanded on the injected side (Figures 4A, C compare NI to MO11). In control explants, the β-catenin protein was present near the plasma membrane and in the nuclei of the cells that are at the leading edge of the explant but was mostly restricted to the plasma membrane in cells within the explant (Figure 4A, NI) (Alfandari et al., 2010). In contrast, explants missing Adam11 show nuclear β-catenin localization all the way through the explant (Figure 4A MO11). In addition, it is also clear that the nuclei of the control explants are more packed (Figure 4A DAPI) than the one from the Adam11 KD, suggesting that the cells are denser on the FN substrate (Figure 4D). Nuclear β-catenin is known to activate gene expression, including *slug* (Figure 3A) and *cyclin D1*. We, therefore, tested the expression of CyclinD1 in CNC explants (Figure 4B). Again, while the expression of CyclinD1 is mostly restricted to the periphery of the CNC explant from the control side, the expression is present throughout the explant from the injected side, and the overall intensity is significantly higher than in the control side (Figure 4E). It is important to note that these images are maximum projections so that protein expression levels should not be affected by their relative Z-position within the explant.

### Loss of Adam11 leads to higher proliferation and early EMT

**FIGURE 5:**
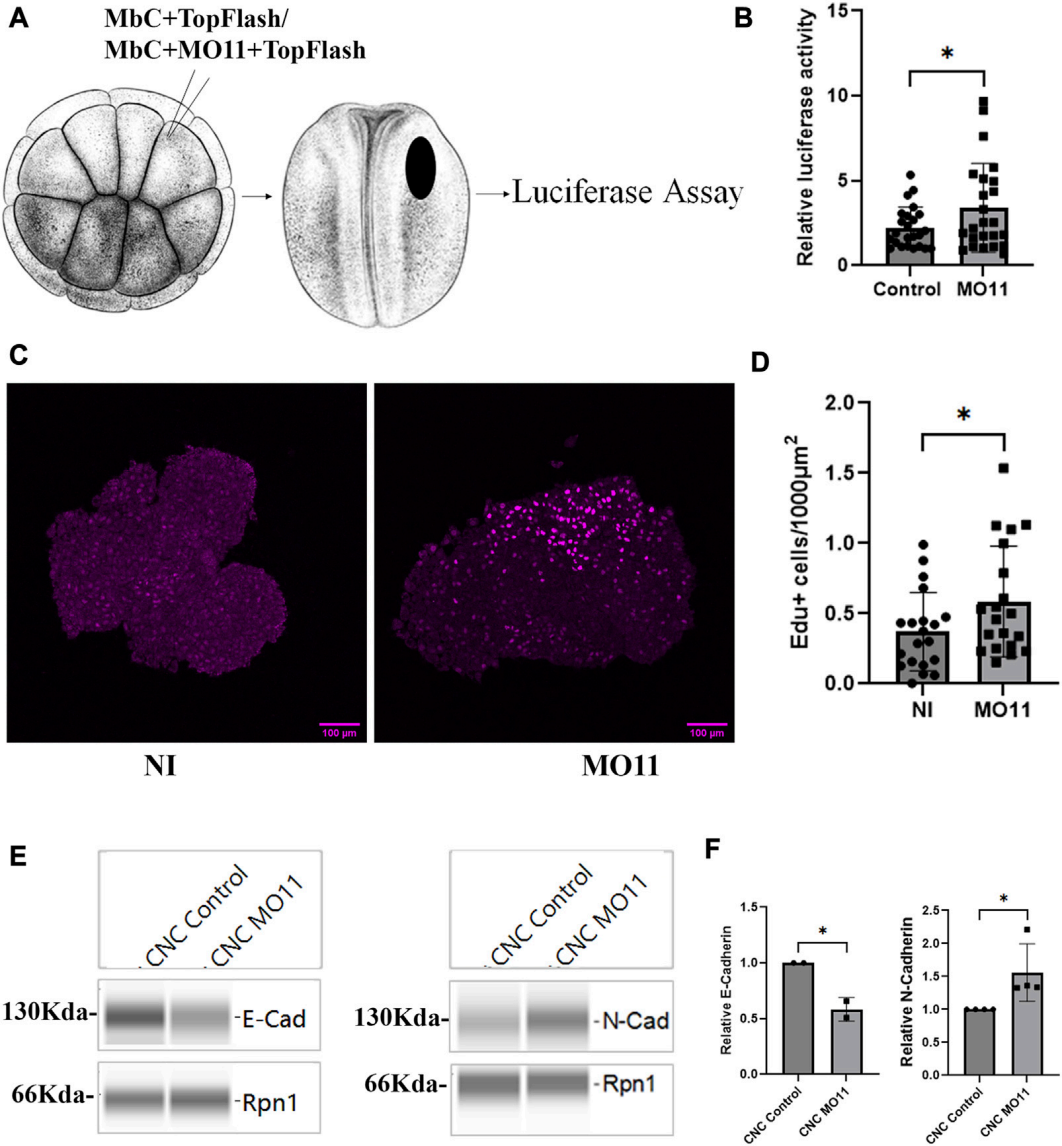
Adam11 KD leads to increase β-catenin transcriptional activity, higher proliferation and increased N-cadherin expression. **(A)** Graphical representation of the luciferase experiment in embryos. 16-cell stage embryos were injected with a lineage tracer and 10 pg of the top flash reporter plasmid together with 2 pg of the CMV renilla plasmid in one dorsal animal blastomere. Embryos with lineage tracer present in the dorsal anterior quadrant (Black oval) at the neurula stage were selected and extracted individually for dual luciferase reporter assay. **(B)** Top flash luciferase reporter activity. Each point represents one embryo from four biological replicates. Control embryos (dots) were compared to embryos injected with MO11 (Square, *t*-test, *=*p* < 0.05). **(C)** Confocal imaging of Edu (magenta) labeled CNC explants 3 h after dissection. Embryos were incubated with Edu at stage 15 for 2 h, and CNC were dissected at stage 17. CNC were allowed to migrate on FN for 3 h prior to fixing. **(D)** Histogram showing the relative number of Edu-positive cells per area. Numbers were normalized to the size of each explant. Dots represent each explant from three biological replicates. **(E)** Capillary Western blot of E-cadherin and N-cadherin from CNC explants dissected at stage 17. **(F)** Quantitative representation of the capillary Western blot showing E-cadherin and N-cadherin protein levels normalized to Rpn1. Student’s t-test was performed for data represented in **(B,D,F)** (*=*p* < 0.05).

To directly test if the β-catenin transcriptional activity was affected by the loss of Adam11, we used the well-characterized Top-Flash luciferase reporter (Chuang et al., 2010; Baarsma et al., 2011) *in vivo*. We targeted the Top flash reporter to the precursor CNC territory at the 16-cell stage (Moody, 1987) in either control embryos or embryo KD for Adam11. We found a statistically significant increase in Top flash activity in embryos KD for Adam11 (Figure 5B).

Given the known role of CyclinD1 in controlling the G1 to S transition (Stacey, 2003), we tested the proliferation of CNC cells using Edu labeling. Embryos were incubated in Edu for 2 hours at the beginning of neurulation (stage 15/16) when the expression of Adam11 mRNA starts accumulating (Session et al., 2016). CNC explants were dissected at stage 17 and allowed to migrate on fibronectin for 3 h prior to fixing and staining for Edu-positive nuclei (Figure 5C). We chose this timing to allow enough cells to divide in the CNC since we only have 300 cells per explant on average (2 h labeling, Fixed 4 h after the end of labeling). We observed a significant increase in Edu-positive cells in CNC with lower Adam11 when compared to control explants (Figure 5D). To account for variability in explant size and labeling stage between experiments, the number of Edu-positive nuclei was normalized to the size of each explant and set to one for the control explants. Loss of E-cadherin and gain of N-cadherin is a classical hallmark of EMT in CNC and cancer (Araki et al., 2011; Scarpa et al., 2015; Huang et al., 2016; Cousin, 2017), we therefore, tested the expression of E-cadherin and N-cadherin via quantitative capillary Western blot on protein extracts from stage 17 CNC explants. When normalized using Ribophorin 1 (Rpn1 as a loading control), we found that CNC lacking Adam11 expressed less E-cadherin and more N-cadherin than the control explants (Figures 5E, F). Together, these results show that loss of Adam11 increases β-catenin transcriptional activity and causes earlier EMT, suggesting that Adam11’s role is to reduce and/or delay this activity in the neural crest.

### Adam11 binds to protein from the BMP4 and Wnt signaling pathways

**FIGURE 6:**
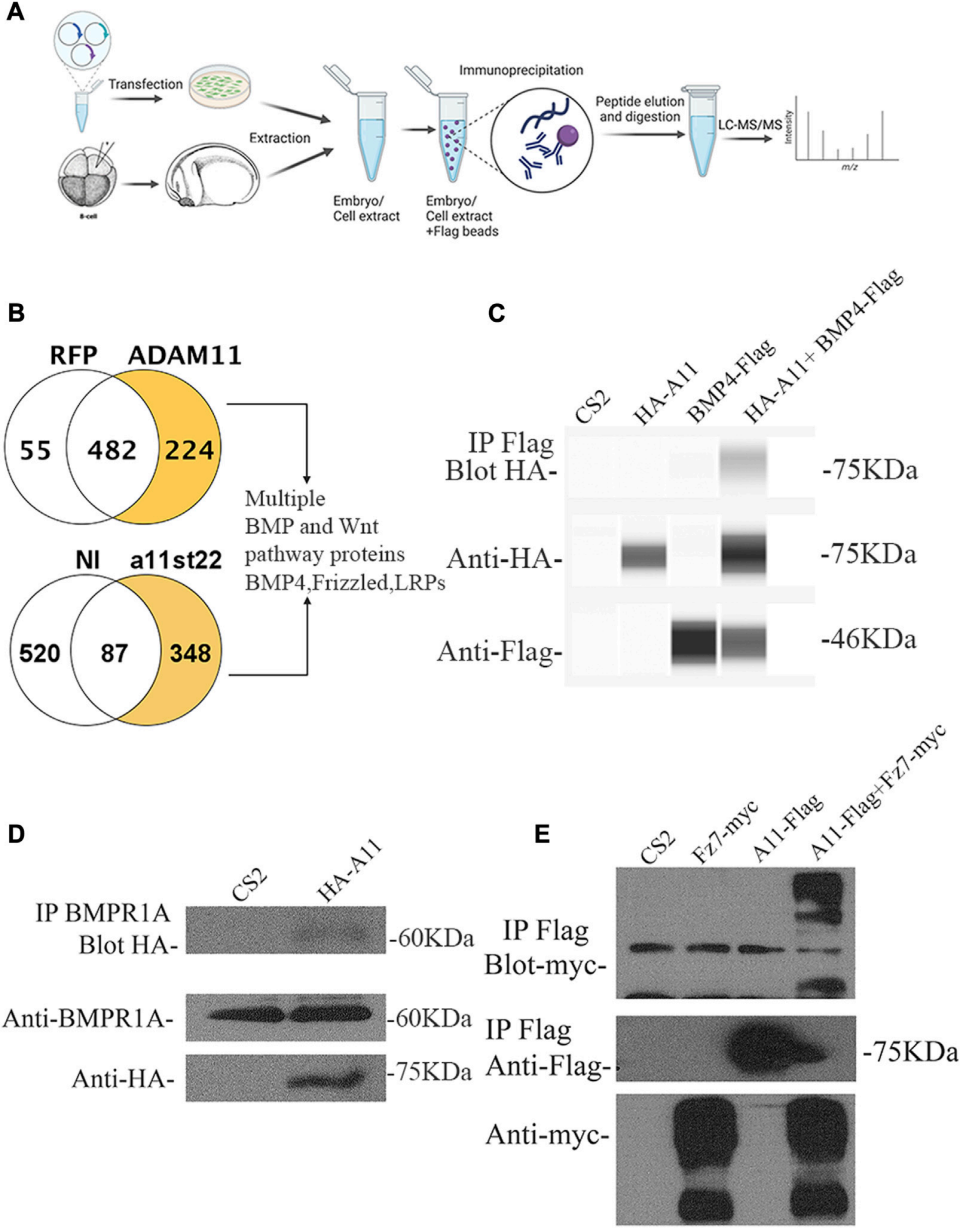
Adam11 binds to proteins of the BMP4 and Wnt signaling pathways. **(A)** Schematic representation of the Lc/Ms/Ms experiment. Both human Hek293T cells and embryos were used to produce Adam11.L-Flag. Hek293T cells were transfected while embryos were injected at the 8-cell stage in a dorsal animal blastomere to target CNC with Adam11.L-flag mRNA or an irrelevant flag-tagged protein (RFP-Flag). The embryos were grown until stage 22 sorted and extracted. The Flag-tagged proteins were immunoprecipitated and subjected to protein digestion and LC/MS/MS. **(B)** Proteins identified in either *Xenopus* embryos (348) or Hek293T cells (224) with at least two unique peptides that were absent in the negative controls were compared. **(C)** BMP4 binding to Adam11.L (HA-A11) was confirmed by co-immunoprecipitation in Hek293T cells. HA-Adam11.L was co-transfected with BMP4-Flag. Immunoprecipitation (IP first line) was performed using an anti-Flag antibody, and Western blot (Blot) was performed with anti-HA antibody. Total extracts blotted with HA (line 2) and with Flag (Line 3) are provided. **(D)** Endogenous human BMPR1A binding to *Xenopus* Adam11.L was confirmed by co-IP in Hek293T cells. HA-Adam11 (HA-A11) or empty CS2 vector were transfected. Immunoprecipitation (IP line 1) was performed using an anti-BMPR1A antibody, and Western blot (Blot) was performed with anti-HA. Total extracts were blotted with anti-BMPR1A (Line 2) and anti-HA (Line 3) antibodies. **(E)** Frizzled7 (Fz7) binding to Adam11 was confirmed by co-IP in Hek293T cells. Adam11-Flag (A11-Flag) was co-transfected with Frizzled7-myc (Fz7-myc). Immunoprecipitation was performed using an anti-Flag antibody (line 1 and 2), and Western blot was performed with anti-myc (Line 1) and anti-Flag (Line 2). Total extracts were blotted with the anti-myc (line 3) antibody.

While the apparent early EMT pointed to β-catenin, we wanted to use an unbiased approach to identify additional functions for Adam11 in neural and neural crest cells. Adam11 is a non-proteolytic ADAM with no known role during embryogenesis. Protein interaction studies have identified putative binding partners for Adam11 in mouse, including integrins and LGl proteins in neurons (Takahashi et al., 2006a; Takahashi et al., 2006b; Sagane et al., 2008; Kole et al., 2015; Wang et al., 2018). These putative protein interactions cannot explain the effect of Adam11 KD on β-catenin activation. In the absence of an antibody to *Xenopus* Adam11 at the time of this experiment, we expressed the Flag-tagged Adam11.L in *Xenopus* Cranial neural crest cells and human Hek293T cells and pulled down interacting proteins (Figure 6). The flag-tag experiment also has the advantages that no part of the proteins is blocked by the antibody. In embryos, we injected at the 8-cell-stage to target the CNC and extracted the proteins at either the neurula or tailbud stages (4 samples from two independent experiments). We considered proteins with at least two unique high-confidence peptides (95% peptide threshold, 99% protein threshold) that were present in at least two of the 4 samples as potential candidates. We also performed Flag pull down on human Hek293T cells transfected with Adam11-Flag or RFP-Flag. Among the proteins that were found in both data sets (Supplementary Table S1), we identified the secreted protein BMP4. We also found multiple members of the LRP family of protein and the Wnt receptor FzD6, but these were not present in both data sets (LRP2 in embryos, LRP5-6, and Fzd6 in Hek293T cells). We also found the BMP4 receptor BMPR1A in Hek293T cells. Given the critical role of the Wnt/β-catenin and BMP4 signaling pathway to both neural and neural crest cell development and our results showing increased β-catenin activity, we focused on these putative binding partners for this study. In addition, other ADAMs have been shown to interact with Wnt receptors or small secreted forms of the receptor that act as antagonists (Esteve et al., 2011; Abbruzzese et al., 2015); thus, we also tested the interaction with Wnt receptor Fzd. We were able to confirm the interaction of Adam11 with BMP4 (Figure 6C) and the BMP receptor 1A (Figure 6D) by co-immunoprecipitation. We also identified the interaction of Adam11 with the Wnt receptor Fzd7, a key component of the β-catenin signaling pathway in embryos (Figure 6E).

### Loss of Adam11 increases Hsp90ab1 expression

**FIGURE 7:**
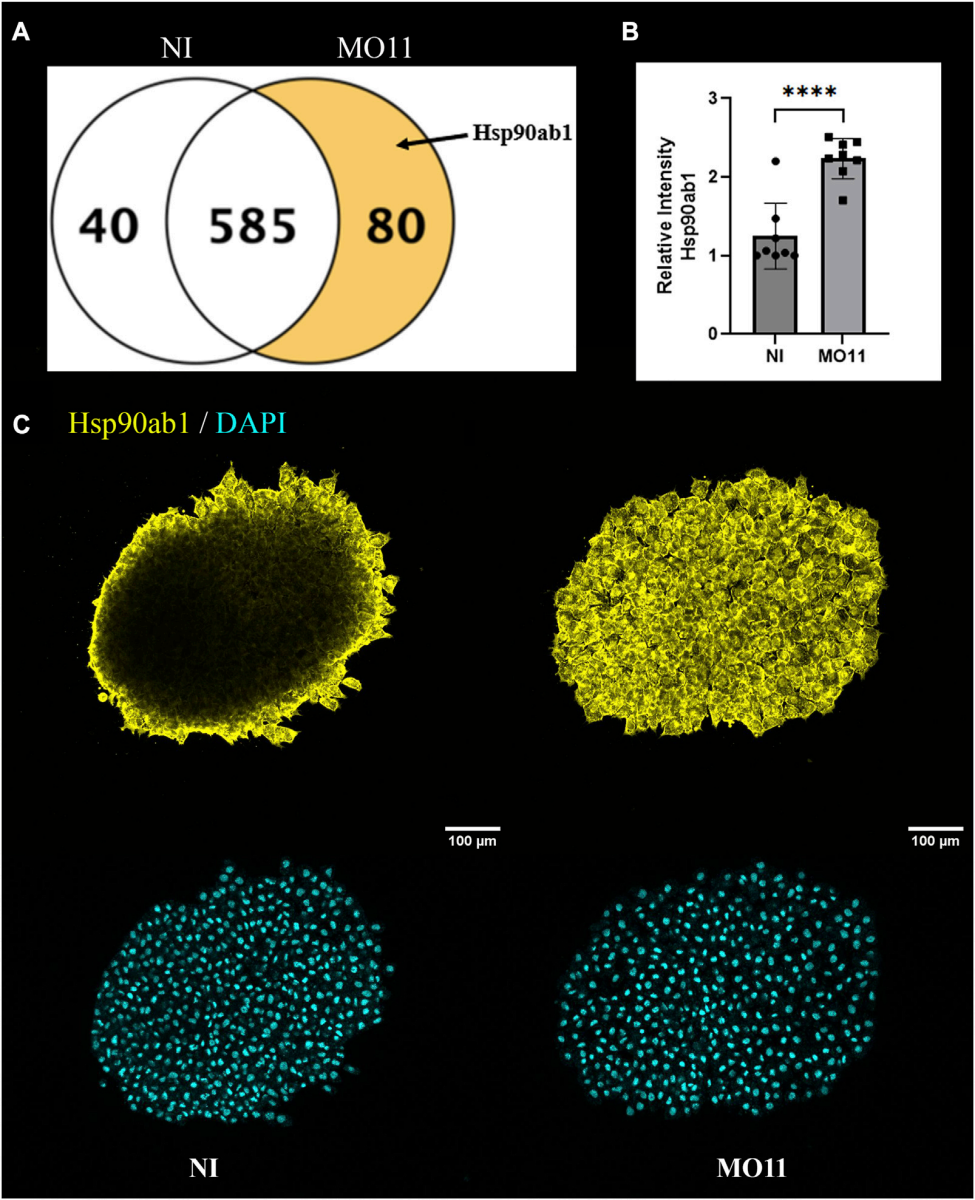
Adam11 KD increases Hsp90ab1 expression. **(A)** Venn diagram of proteins with a minimum of two peptides statistically downregulated (40) or upregulated (80) in CNC explants from Adam11 KD (MO11) compared to non-injected (NI) control CNC. **(B)** Relative fluorescence intensity of Hsp90ab1 in CNC explants shown in **(C)**. The dots represent the average intensity for each explant from three biological replicates. **(C)** Confocal imaging of CNC explants 1 hour after dissection. CNC explants were dissected at stage 17 and placed on a fibronectin-coated glass coverslip. Explants were stained for Hsp90ab1 (yellow) and DAPI (Cyan). Student’s t-test was performed for statistical analysis (****=*p* < 0.0001).

To understand better how the Adam11 KD changed the state of the CNC cells, we performed mass spectrometry on the dissected CNC explants (Figure 7A). After analysis of triplicate experiments, we focused on proteins that were significantly up or downregulated and identified with a minimum of one (Supplementary Figure S1; Supplementary Table S2; Supplementary Table S3) or two (Figure 7A) high confidence peptides. Selecting one peptide rather than two reduces the confidence that the protein is present but allows to identify proteins that are less abundant. While the majority of proteins identified were common (386 at 2 peptides and 2020 at 1 peptide), we found 80 proteins that were upregulated in the morphant and 40 proteins that were downregulated in the CNC KD for Adam11. In particular, we found that Hsp90ab1 was only detected in Adam11 KD CNC. Hsp90ab1 has been shown to interact with the β-catenin destruction complex (Cooper et al., 2011) as well as LRP5 (Wang et al., 2019) protein to increase β-catenin signaling and promote EMT. It is important to note that HSP expression is not a general response to Morpholino as only nine HSP proteins were identified in our MS, three were upregulated (Hsp90ab1, Hsp90b1 and Hspa4). In addition, Hsp90b1 and Hspa4 were also upregulated in CNC KD for Adam13/33 but not Hsp90ab1 showing a clear specificity (Supplementary Figure S2). To confirm the result from the Mass spectrometry experiments, we performed immunofluorescence on CNC explants using an antibody to Hsp90ab1 (Figures 7B, C). These results are strikingly similar to those observed for CyclinD1 (Figure 4B) and confirm the upregulation of Hsp90ab1 in CNC lacking Adam11.

### Adam11 increases BMP4 signaling

**FIGURE 8:**
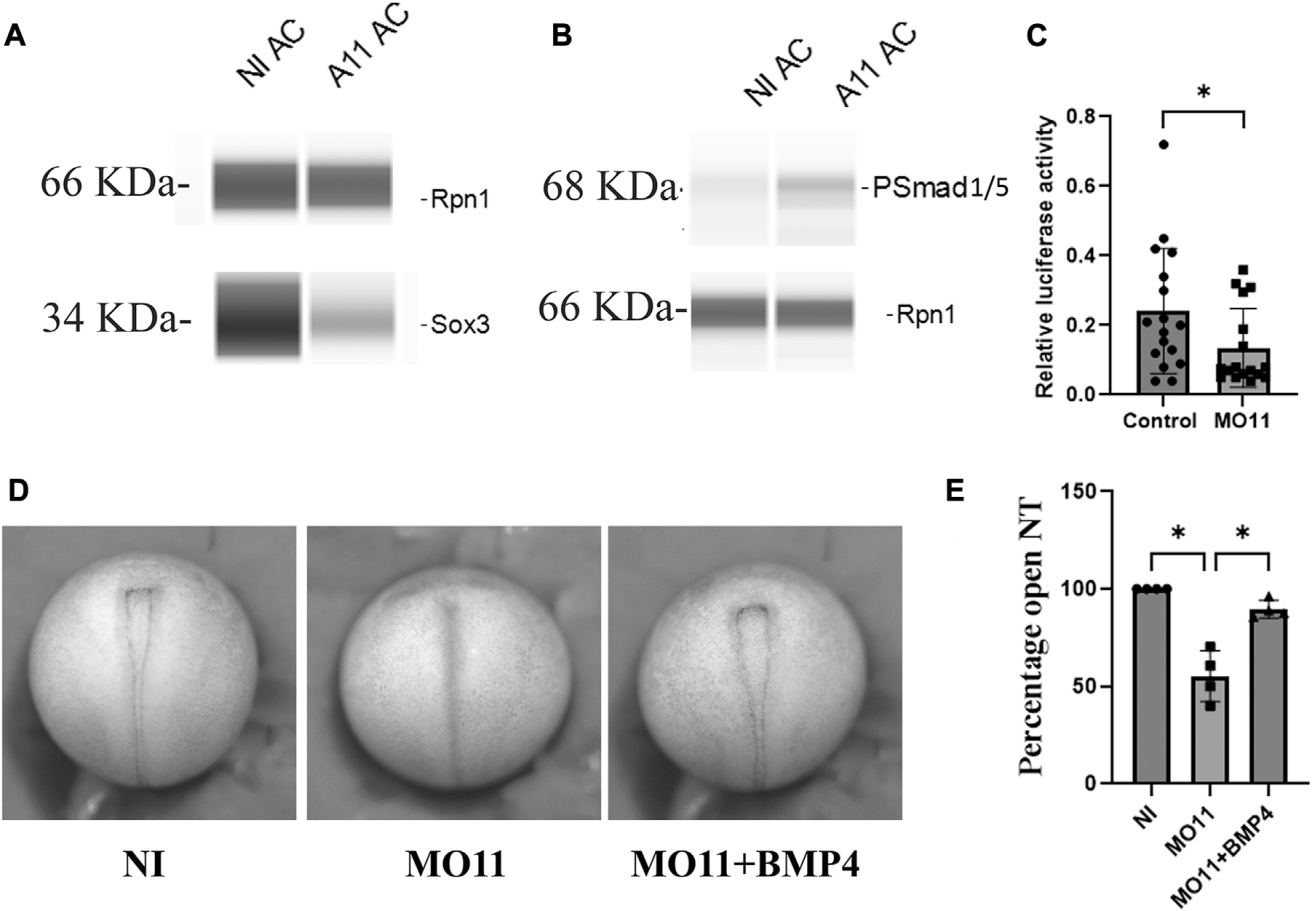
Adam11 increases BMP4 signaling. **(A,B)** Representative capillary Western blot. Animal caps from control embryos or embryos injected with Adam11 mRNA (1 ng) were dissected at stage 9, dissociated in CMF for 2 h and reassociated, and grown in Danilchick media until sibling embryos reached stage 20. Proteins were then extracted and analyzed by capillary Western blot using antibodies to ribophorin1 (rpn1) as a loading control and either Sox3 to detect neural induction **(A)** or phospho-smad1/5 antibody to measure BMP4 signaling activity **(B)**. **(C)** Histogram representing the relative luciferase activity between control and Adam11 KD. Embryos were injected at the 8-cell stage with 1.5 ng of MO11, 20 pg of pGL2-15xGCCCG-lux BMP reporter, and 4 pg of CMV renilla (*=*p*< 0.05). **(D)** Representative photograph of neurula stage embryos (Dorsal view anterior up). Embryos were injected at the one-cell stage with either MO11 or MO11 and 12.5 pg of BMP4 mRNA. **(E)** Histogram representing statistical analysis of the phenotype represented in **(D)**. Asterisk represents the statistical significance at *p* < 0.05 (*).

As shown above, Adam11 interacts with both BMP4 and its receptor BMPR1A (Figure 6). We also found that secreted BMP4 interacted with shed Adam11 in the conditioned media of co-transfected cells (data not shown). We, therefore, tested if Adam11 binding to BMP4 increased or decreased BMP4 signaling. For this, we used the animal cap (AC) assay. The intact animal cap represents a naive ectoderm with high BMP4 signaling and no expression of Adam11 based on published RNAseq data (Xu et al., 1995; Sive et al., 2007; Haas et al., 2019). Dissociation of the animal cap dilutes BMP4 signaling and induces neural tissue that expresses a high level of Sox3 (Figure 8A). We repeated these experiments using AC expressing injected Adam11.L. Following animal cap dissociation into single cells, we found that Sox3 was significantly reduced in explants from embryos overexpressing Adam11 (Figure 8A, A11AC). In the same samples, we found that the phosphorylated Smad1/5 was increased by Adam11 overexpression (Figure 8B), consistent with an activating role of Adam11 toward BMP4. To further test the activity of Adam11 towards BMP4 signaling during neurulation, we performed a luciferase assay using the pGL2-15xGCCCG-lux BMP reporter (Alkobtawi et al., 2021) targeted at the 8-cell stage (Figure 8C). The luciferase activity was decreased with Adam11 KD further confirming that Adam11 increases BMP4 activity. BMP4 plays a critical role during neural induction by defining the ventral limit of the neural tissue, while BMP4 inhibitors are essential for neural induction (Weinstein and Hemmati-Brivanlou, 1997). To test if Adam11 interaction with BMP4 was important for the neural tube closure phenotype (Figure 1D), we repeated the assay using low doses of BMP4 mRNA (12.5 pg) to rescue the neural tube closure phenotype (Figures 8D, E). Indeed, injection of BMP4 in the dorsal part of the embryo rescued the neural tube closure timing to a level indistinguishable from the non-injected control (Figure 8E). Taken together, these results show that Adam11 interacts with the BMP4 ligand and receptor to increase local signaling to a level critical for proper neural tube closure. The role of Adam11 in controlling BMP4 signaling levels is also consistent with our observation that Adam11 KD increases the domain of expression of neural markers such as *sox2* and Sox3 (Figure 3).

### Adam11 expression in cancer

**FIGURE 9:**
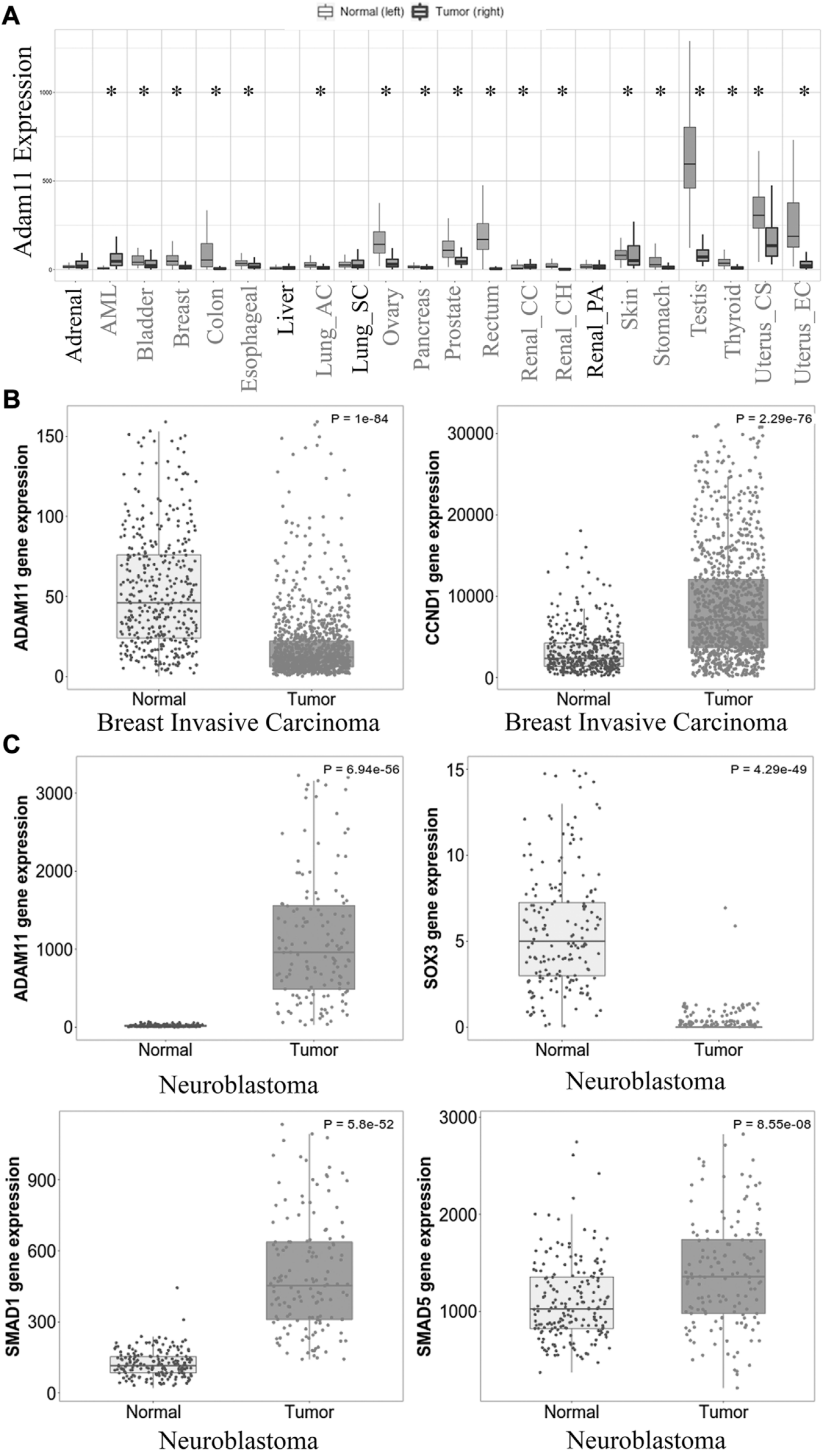
*ADAM11* expression in cancer and cancer metastasis. **(A)** Tnm plot analysis of *ADAM11* expression levels between normal tissue samples and tumors pan-cancer from human patients. Tumor types with significant changes in *ADAM11* expression are indicated with an asterisk. **(B)** Tnm plot analysis of *ADAM11* and *CYCLIND1* expression between multiple normal (403) and tumor (1,097) breast invasive carcinoma patient samples. (*p* = 10^−54^ and *p* = 2.29 10^−76^ respectively). **(C)** Tnm plot analysis of *ADAM11* (*p* = 5.94 10^−56^), *SOX3* (*p* = 4.29 10^−49^), *SMAD1* (*p* = 5.8 10^−52^), and *SMAD5* (*p* = 8.55 10^−5^) expression between Neuroblastoma (149) and normal (190) patient samples. Mann-Whitney test was performed for the *p*-values indicated in the legend. The *Y*-axis for figure **(B)**, **(C)** represents normalized counts for the gene mentioned.

We have shown that *Xenopus* Adam11 restricts β-catenin activity in the CNC. Loss of Adam11 increases single CNC cell migration *in vitro,* resembling an epithelium to mesenchyme transition (EMT). Given the role of β-catenin in cancer and the known relation between EMT and cancer metastasis, we looked at human *ADAM11* gene expression in available databases (Bartha and Gyorffy, 2021). Out of 56,938 unique multilevel quality-controlled samples, *ADAM11* expression was found to be significantly different in 18 out of 23 tumor types (Figure 9A*). It was significantly reduced in 16 of the 18 tumor types. Given the previous observation that *ADAM11* was mutated in two breast cancer samples (Emi et al., 1993), we further focused our analysis on data obtained from invasive breast carcinoma (Figure 9B). The expression level was significantly lower in the samples from cancer patients (1,097 patients) when compared to normal tissues (403 patients, mean FC = 0.35, *p* = 10^−84^). Interestingly in the same samples, *CYCLIND1* was found to be significantly increased (mean FC = 4.15, *p* = 2.29 10^−76^). Together, these results strongly suggest that Adam11’s ability to restrict β-catenin activity and delay EMT in the CNC is conserved in adult tissues and that loss of ADAM11 may contribute to β-catenin hyperactivation and *CYCLIND1* overexpression, leading to tumor progression and metastasis.

One type of tumor in which *ADAM11* is increased rather than decreased is neuroblastoma (Figure 9C, 190 normal and 149 tumor samples, mean FC = 61.52 *p* = 6.94 10^−56^). In these tumors, we found that *SMAD1* and *SMAD5* were significantly increased (mean FC = 4.46 *p* = 5.8 10^−52^ and FC = 1.31 *p* = 8.55 10^−8^ respectively) while *SOX3* was significantly decreased (mean FC = 0.13 *p* = 4.29 10^−49^). An observation that is consistent with our results showing that the overexpression of Adam11 in the naïve animal cap ectoderm increases BMP4 signaling, resulting in increased Smad1-5 phosphorylation and decreased Sox3 (Figure 8), while loss of Adam11 increases Sox3 expression (Figures 3E, F). It is important to note that for human cancer, we do not have phosphorylation data concerning Smad and that the increase in mRNA expression, while indicative, is not a direct indicator of an increase in BMP4 signaling.

### Reduction of ADAM11 in B16 melanoma increases cell proliferation

**FIGURE 10:**
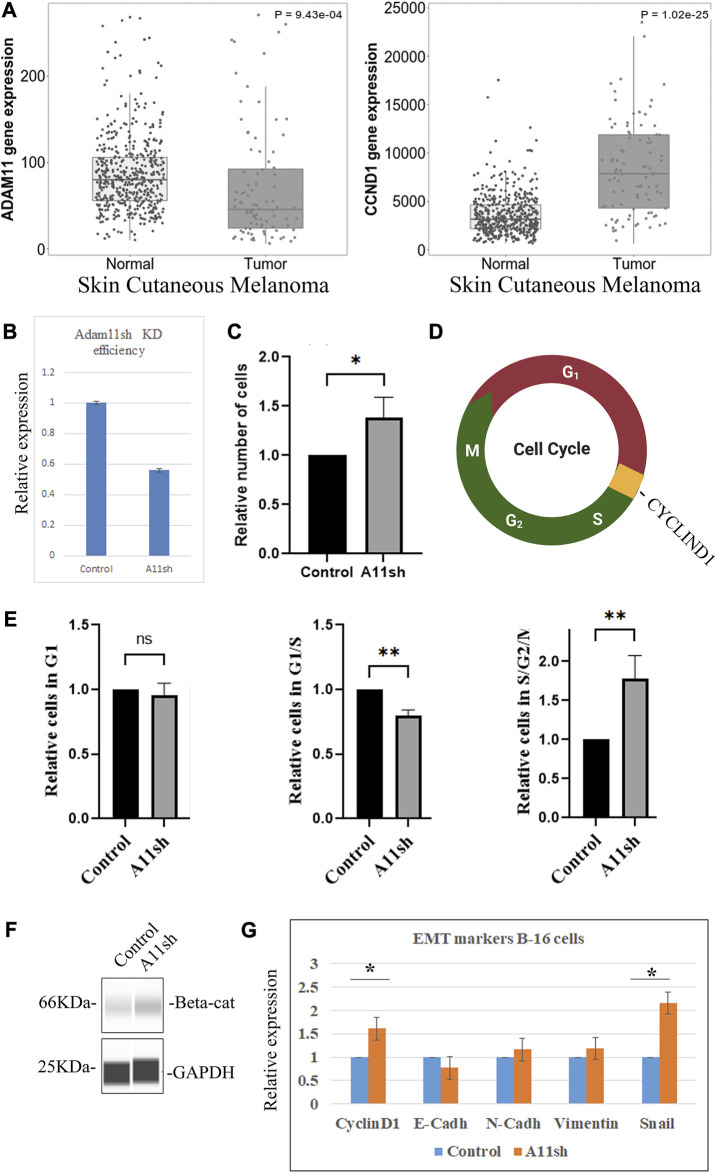
ADAM11 expression in Melanoma. **(A)** Tnm plot analysis of *ADAM11* (*p* = 9.43 10^−4^) and *CYCLIND1* (*p* = 51.02 10^−25^), expression between multiple normal (474) and (103) skin cutaneous melanoma tumor patient samples (*Y*-axis represents normalized counts for the gene mentioned). **(B)** RT-qPCR analysis of mouse B-16 melanoma cells transfected with either empty plasmid control or shRNA to mouse Adam11 (A11sh). Cells were harvested for mRNA and processed for RT-qPCR. *Adam11* levels between the samples was normalized to *Beta-actin.* **(C)** Histogram showing the relative number of cells present 48 h post-transfection in control and shA11 wells. **(D)** FUCCI cell cycle reporter. Cells transfected with the FUCCI reporter fluoresce in Green during the S, G2, and M phase, in Red during G1 and Yellow (both red and green) during the G1/S transition. **(E)** Histogram depicting the relative number of cells in each phase of the cell cycle following silencing of mouse Adam11 (A11sh). The relative number of cells in each phase was set to one for the control. Cell numbers were obtained by FACS analysis (50,000 cells counted per experiment, three independent experiments, **=*p* < 0.01). As expected, given the regulation of CyclinD1, significantly fewer cells were found in the G1 to S transition and more cells in S, G2, and M. **(F)** Capillary Western blot of B-16 melanoma cells transfected with either empty plasmid control or Adam11sh (A11sh) plasmid, probed with β-catenin and Gapdh antibodies. **(G)** RT-qPCR analysis of B-16 melanoma cells transfected with either empty plasmid control or A11sh plasmid. *Cyclind1, E-cadherin, N-cadherin, Vimentin, and Snail* levels between the samples was normalized to *Beta-Actin.* The fold change calculated using the ΔΔCT is indicated. Error bars represent the standard deviation (* means *p* < 0.05).

The human cancer analysis indicates a strong correlation between *ADAM11* expression and *CYCLIND1* levels. To further test if *Adam11* expression in cancer cells could modulate the Wnt signaling pathway, we used mouse B16 melanoma cells (Poste et al., 1980). These cells are derived from neural crest cells, express a relatively high level of *Adam11*, and are highly invasive *in vivo* when injected into the bloodstream but not subcutaneously. Furthermore, in skin cutaneous melanoma, published RNA-seq data shows a decreased level of *ADAM11* and an increased level of *CYCLIND1* between tumors (103 patients) and normal samples (474 patients, Figure 10A). We used shRNA mediated inhibition of Adam11 in these cells to test the effect on cell proliferation. Based on the CNC results, we expected that loss of Adam11 would increase Wnt signaling and CyclinD1 expression (Figure 4), resulting in increased cell proliferation (Figure 5). While we obtained only a modest but reproducible reduction in *Adam11* expression (35%, Figure 10B), we were able to see an increase in total cell number (40%) in cells with reduce Adam11 expression (Figure 10C). In order to understand the increase in cell number (Figure 10D), we used the well-characterized FUCCI cell cycle reporter (Sakaue-Sawano and Miyawaki, 2014), transfected in the context of Adam11KD and used FACS to follow cell cycle progression. Following successful transfection, the cells are red when they are in G1, yellow during the transition from G1 to S, or green if they are in S, G2, or M phase (Figure 10D). We found that the decrease in *Adam11* resulted in a significant decrease in the number of cells in the G1-S transition and increased number of cells in S, G2 and M phases (Figure 10G). Like our finding in CNC, we found that B16 with reduced Adam11 have increased β-catenin (Figure 10F). We then tested by RT-qPCR the expression of multiple markers of EMT and target of β-catenin. We observed a statistically significant increase in *CyclinD1* and *Snail* levels (Figure 10E), and while there was a small decrease in *E-cadherin* and an increase in *N-cadherin* and *Vimentin* that fit with our EMT expectation, those were not significant.

## Discussion

Adam11 is a member of the non-proteolytic ADAM that appears essential for the proper function of the central nervous system in mammals (Takahashi et al., 2006a; Takahashi et al., 2006b). In addition, ADAM11 was identified as a putative tumor suppressor 30 years ago, but no additional data in support of this hypothesis was ever published (Emi et al., 1993). Here, we show that Adam11 influences Wnt and BMP4 signaling pathways during neurulation and neural crest cell migration. Loss of Adam11 increases β-catenin transcriptional activity and decreases BMP4 signaling. Loss of Adam11 accelerates neural tube closure and accelerates the transition from collective to single-cell migration in the CNC. We further show that Adam11 expression is reduced in multiple tumors. We propose that Adam11 functions as a regulator of Wnt and BMP4 signaling to control the expression of neural and neural crest differentiation markers as well as the onset of EMT and morphogenetic movement in the embryo.

### ADAM11 in cancer

ADAM11 is well conserved between *Xenopus*, Mouse, and Human both in the extracellular and cytoplasmic domains (Supplementary Figure S3). This conservation is especially striking in the short cytoplasmic domain that does not contain any putative functional attribute. The least conserved region is the pro-domain, which in proteolytic ADAM controls the activation. Thirty years ago, ADAM11 was found to be mutated in two mammary gland tumors and was postulated to be a tumor suppressor (Emi et al., 1993). Surprisingly, no other study tested this hypothesis. Given our results showing that Adam11 regulates β-catenin activity in *Xenopus* embryos (Figure 4), we analyzed the expression of *ADAM11* in available tumor expression data. The analysis shows that *ADAM11* expression is significantly lower in most solid tumors (Figure 9A), including the neural crest-derived melanoma, and this is even more pronounced in invasive breast carcinoma, a type of tumor where ADAM11/MDC was originally found mutated, supporting the hypothesis that *ADAM11* may be a tumor suppressor gene. We find that in addition to the significant decrease in *ADAM11* (Figures 9, 10), there was also a significant increase in *CYCLIND1*. While there is no causal relation in this observation, our data from *Xenopus* embryos and mouse B-16 melanoma cells shows that decreasing Adam11 protein level is sufficient to increase β-catenin activity and CyclinD1 expression, suggesting a conserved function of Adam11 in cancer. It will be of interest to test if human ADAM11 can also regulate β-catenin and rescue neural tube closure timing and CNC migration in *Xenopus* embryos. On the other hand, it would be interesting to test if increasing ADAM11 protein levels in cancer cells is sufficient to reduce β-catenin activity, *CYCLIND1* expression, and cell proliferation. While this seems like an interesting strategy, we have also found that ADAM11 expression is increased in Neuroblastoma, a childhood cancer thought to be derived from neural crest cells. In Neuroblastoma, the increase in ADAM11 expression is associated with increased expression of Smad1 and 5 (Here mRNA expression rather than Phosphorylation) and decreased expression of Sox3, similar to what we observed when overexpressing ADAM11 in naïve ectoderm in *Xenopus* embryos (Figure 8) suggesting that in Neuroblastoma ADAM11 positive regulation of BMP4 signaling could lead to the repression of neural differentiation markers (Sox2 and 3) resulting in more naïve cells. These naïve or “stem” cells could contribute significantly to the malignancy. Therefore, our observation suggests that a fine control of ADAM11 expression is critical for normal development as well as tissue homeostasis. A careful structure/function analysis will be required to understand the role of each of the functional domains to the overall function of ADAM11.

### Neural crest cell migration

We have found that CNC migrated well *in vitro* but not *in vivo*. This is certainly not unique, as we found this to be the case in most ADAM inhibition, highlighting the essential role of ADAM interaction with the extracellular environment (McCusker et al., 2009; Cousin et al., 2012). For example, we have previously shown that Adam13/33 activity is no longer required when the CNC cells are physically separated from the underlying mesoderm *in vivo* (Cousin et al., 2012). *In vitro*, CNC migrates on top of a pure fibronectin substrate, while *in vivo,* the CNC are in a three-dimensional extracellular matrix that is crosslinked and composed of multiple proteins in addition to fibronectin (e.g., Laminins and collagens). In addition to this difference in structure, *in vivo*, CNC are presented with both attractive and repulsive signals that are absent *in vitro*.

While β-catenin is essential for proper CNC induction, studies have shown that β−catenin activity needs to be reduced for proper migration and that abnormal activation within the CNC leads to inhibition of migration (Maj et al., 2016). This suggests that the excess activation of β−catenin following Adam11 KD could be responsible for the perturbation of CNC migration *in vivo*. In contrast, both chemical inhibition of GSK3 and activation of TcF3 also inhibit migration *in vitro,* preventing single-cell dispersion (Maj et al., 2016). This is opposite to the phenotype that is observed in the Adam11 morphant embryos where single cell number increase, and both speed and persistence are higher. This suggests that the loss of Adam11 is not simply a hyperactivation of β−catenin and that the decrease in BMP4 signaling may also influence CNC migration. Certainly, the activation of β−catenin following the loss of Adam11 is much more subtle as the overall level of the protein is unchanged, and only the nuclear localization is increased.

Adam11 knock-out mice show normal mechanical and noxious heat responses but have significantly decreased chemical nociception, indicating that Adam11 is necessary for the formation or the relay of specific set of sensory information (Takahashi et al., 2006a). The sensory neurons are derived mostly from the cranial placodes and cranial neural crest (cranial ganglia) anteriorly and from the trunk neural crest posteriorly (Vermeiren et al., 2020). The loss of specific type of nociception in Adam11 knock-out mice (Takahashi et al., 2006a) raises the possibility that the defect in these neurons might be a consequence of abnormal specification or migration of the neural crest. Adam11 transcripts have been detected in the dorsal root ganglia and the neural crest in the mouse (Rybnikova et al., 2002; Diez-Roux et al., 2011) and the frog (Cai et al., 1998). While no obvious anomalies were reported in the DRG of Adam11 KO mice, it is likely that the absence of the small nociceptive neurons would not be detected without using cell-specific markers. Alternatively, it is also possible that these neurons exist but fail to properly organize their synapse, as shown for the cerebellar basket cell (Kole et al., 2015). Further analysis of the Adam11 morphant embryos at a later stage could help identify subtle DRG defects.

### Neural tube closure

Neural tube closure is a complex morphogenetic event that involves hundreds of different genes. In *Xenopus*, like in mammals, the neural folds elevate toward the center of the neural plate and then fuse. It is important to note that the neural plate, as defined by Sox3 expression, is wider on the side missing Adam11. Defects in the non-canonical Wnt planar cell polarity (PCP) pathway induce a wider neural plate, but contrary to Adam11 KD, the neural fold fails to reach the midline (Goto and Keller, 2002; Wallingford and Harland, 2002). While the role of the PCP pathway is clearly critical, β-catenin has been shown to regulate the expression of Pax3 and Cdx2, two transcription factors critical for neural tube closure. In the absence of β-catenin, neural tube closure is inhibited but can be rescued by the expression of Pax3 (Zhao et al., 2014). Therefore, an increase or early activation of β-catenin in Adam11 morphant embryos could lead to the activation of Pax3 and the early neural tube closure. In mouse, FGF3 mutants have elevated levels of BMP signal, increased neural cell proliferation, and delayed neural tube closure (Anderson et al., 2016). This would be consistent with the decreased BMP4 signaling and accelerated neural tube closure in Adam11 morphant embryo and could explain the rescue observed using low levels of BMP4 mRNA (Figures 8D, E). Careful analysis of β-catenin and BMP4 signaling levels prior to neural tube closure would be critical to determine if they are indeed responsible for this Adam11 phenotype. In the future, it would be interesting to determine if the early closure of the neural tube affects the proper specification of the various neuronal cell types and their organization. This could partly explain the neurological disorder observed in mice lacking Adam11. It is clearly much more difficult to evaluate neural tube closure timing in mouse embryos, and as such, it is understandable that those have not been reported.

### Model

**FIGURE 11:**
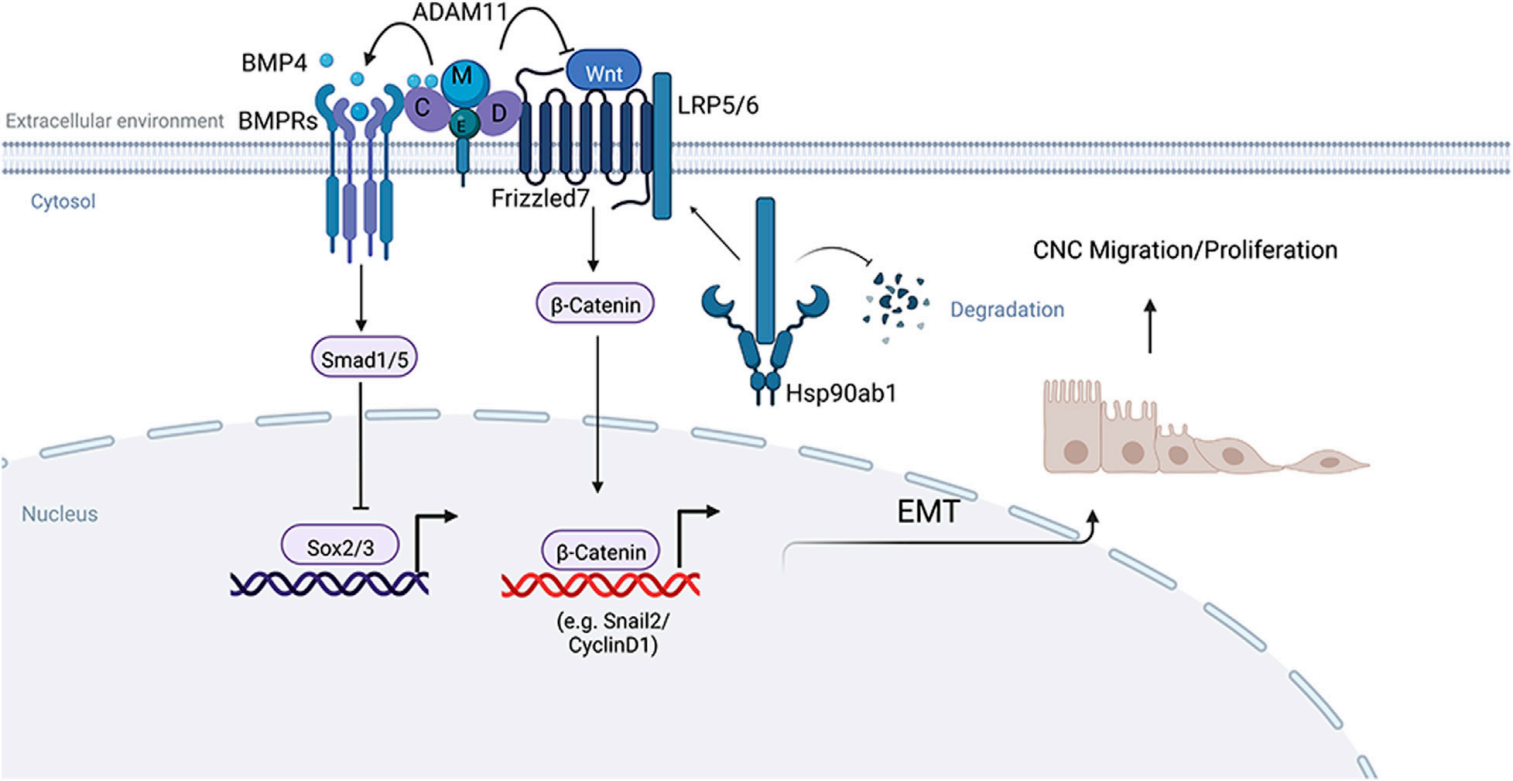
Model of Adam11 function. Adam11 binds to BMP4 and Frizzled on the plasma membrane to increase BMP4 and decrease Wnt/β-catenin signaling. The result of the increased BMP4 signaling is the inhibition of the neural markers Sox2 and Sox3. The result of the decrease in β-catenin activity is a reduction in CyclinD1 and Slug/Snail2, leading to less cell proliferation and delayed EMT. Loss of Adam11 in CNC leads to an increase in Hsp90ab1 that is known to stabilize Lrp5/6 proteins, thus increasing the Wnt receptor signal and β-catenin translocation to the nucleus. Given the interaction of Adam11 with Fzd and Lrp, it is likely that the increase in Hsp90ab1 is a feedback loop reinforcing the pathway rather than the initial trigger of β-catenin activation. Adapted from “Signaling Pathways Underlying EMT and EndMT: Emerging Roles in Age-related Macular Degeneration (AMD)”, by BioRender.com (2022). Retrieved from https://app.biorender.com/biorender-templates.

Model of ADAM11 function in Cancer and Neural crest cells (Figure 11). We have shown that Adam11 binds to BMP4 and its receptor BMPR1A (Figure 6). Our results using the animal cap dissociation experiment (Figure 8) show that Adam11 increases BMP4 signaling, possibly by retaining BMP4 at the plasma membrane and presenting the protein to the receptor (Figure 11). Thus, Adam11 expression at the surface of the CNC would allow them to amplify the gradient of BMP4 locally restricting the expression of neural markers such as Sox2 and Sox3 to define the CNC zone (Figure 3). At the same time, Adam11 can also bind to Wnt receptors Fzd as well as multiple LRP proteins (Figure 6). We propose that the interaction of Adam11 with these Wnt receptor and co-receptor reduce their capacity to either bind to the Wnt ligand or to induce the cytoplasmic cascade that inhibit the destruction complex of β-catenin (Figure 11). Given the capacity of several ADAM protein to bind to the cysteine rich domain of Fzd or Sfrp proteins (Esteve et al., 2011; Abbruzzese et al., 2015), the usual binding site of the Wnt ligand (Dijksterhuis et al., 2015), Adam11 could simply act as a competitive inhibitor of the Wnt/Fzd complex. Alternatively, Adam11 could wedge itself between the Fzd and LRP proteins reducing the activation of the complex. In CNC lacking Adam11, we find a much higher Hsp90ab1 protein expression. This protein has been shown to stabilize Lrp proteins, thus reinforcing the Wnt signaling pathway. Refining the composition of the Adam11 protein complex and the binding sites of each protein will be required to understand the exact mechanism of Adam11 inhibition of β-catenin activation.

## Materials and methods

### Antibodies

The following antibodies were used in this study: monoclonal antibody to Ribophorin-1 (Rpn1) was used as loading control, RRID:AB_2687673 (Khedgikar et al., 2017). The rat monoclonal to flag tag (Agilent Cat# 200474, RRID:AB_10597743). The monoclonal to *X. laevis* Sox3 (DSHB Cat# DA5H6, RRID:AB_2876376) was developed in the laboratory (Horr et al., 2023) against the full length *X. laevis* protein produced in Hek293T cells. It has been characterized by Western blot and immunofluorescence (R24OD21485). The rabbit polyclonal to HA tag (Applied Biological Materials Cat# G166, RRID:AB_2813867). The Rabbit monoclonal to phospho-Smad 1/5 (Cell Signaling Technology Cat# 9516, RRID:AB_491015). Mouse monoclonal to BMPR1A (Thermo Fisher Scientific Cat# MA5-17036, RRID:AB_2538508). Rabbit polyclonal to β-catenin (Abcam Cat# ab16051, RRID:AB_443301). Rabbit polyclonal to Cyclin-D1 (Bioss Cat# bs-0623R, RRID:AB_10856925). Mouse monoclonal to Hsp90ab1 (Thermo Fisher Scientific Cat# 37-9400, RRID:AB_2533349). The anti-myc antibody 9E10 (DSHB Cat# 9E 10, RRID:AB_2266850). Rat monoclonal against N-cadherin (DSHB Cat# MNCD2, RRID:AB_528119). Mouse monoclonal to *X. laevis* Adam11.L (3C5) was developed in the lab by injecting a bacterial fusion protein containing the DC (disintegrin and cystein rich) domain and isolating the clone by the methods described in (Horr et al., 2023).

### Morpholinos and DNA constructs

Morpholino antisense oligonucleotides (Gene Tools, Philomath OR) were designed to either block the translation of Adam11L and S (MO11 Figure 1, CAT​CAT​TCA​TAC​ATA​ATC​ATT​CAG​C) or the splicing of Adam11L (MO11spl, CCA​ATG​GAG​GCA​TAG​TGG​CAC​TG). Adam11.L was cloned in pCS2+ using cDNA made from RNA extracted from stage 18 X*enopus laevis* embryos. Flag and HA tags were added using Takara infusion cloning according to the manufacturer’s instructions. BMP4-Flag in pCS2 was a generous gift from Dr. Chenbei Chang (University of Alabama). Top Flash was previously described in (Chuang et al., 2010). All constructs were sequenced and transfected in 293T cells to validate protein translation, size and proper cell compartment expression.

### Injections and microdissections

SP6 polymerase was used for capped mRNAs synthesis after digesting using Not1 (Cousin et al., 2011). Injectors were calibrated using a 1 µL capillary needle (Microcaps, Drumond, PA, United States). The injection pressure was set at 15 psi and the injection time set between 50 and 200 ms to obtain a 5 nL delivery. Embryos were injected at 1-cell, 2-cell, 8-cell and 16-cells as described previously (Khedgikar et al., 2017). Embryos were raised at 14°C–15°C until scoring for neural tube closure or CNC migration. For each injection, percentage of open NT was normalized to control embryos and was set to 100%, same was done for CNC migration using Membrane Cherry injected embryos. CNC grafts were performed as described (Alfandari et al., 2001; Cousin, 2018). CNC explants migration assays were performed as described in (Alfandari et al., 2003). All experiments were performed at least three times using different females to determine statistical significance.

### Cell culture and transfection

Hek293T (ATCC, CRL-3216 RCB2202) cells were cultured in RPMI media supplemented with 10 U/mL Pen/Strep, 2 mM L-glutamine, 0.11 mg/mL sodium pyruvate, and 10%FBS (Hyclone, South Logan, UT). Transfections were performed using Polyethylenimine (PEI, Polysciences Inc.). For each transfection, 1 µg of DNA was mixed with 10 µg of PEI in 200 µL of Optimem media (Hyclone) at room temperature for 15 min prior to adding to the cells dropwise. The media was changed after 24 h post transfection.

Mouse melanoma B16/F10 (CRL-6475) cells were a generous gift from Dr. Leonid pobezinsky, the cells were cultured in DMEM media with 10 U/mL Pen/Strep and 10%FBS. Transfections were performed as described for Hek293T.

### Animal cap assays

Animal caps dissociation and reaggregation was done as described in (Sive et al., 2007). Briefly, 10 to 20 animal caps were dissected in 1XMBS (1XMBS: 88.0 mM NaCl, 1.0 mM KCl, 2.4 mM NaHCO_3_, 15.0 mM HEPES [pH 7.6], 0.3 mM CaNO_3_-4H_2_O, 0.41 mM CaCl_2_-6H_2_O, 0.82 mM MgSO_4_) containing 50 μg/mL of gentamycin. They were then transferred to Calcium, Magnesium free MBS (CMF) supplemented with 1 mg/mL of BSA for 2 h with gentle rocking to dissociate the animal cap into single cells suspension. The cells were then transferred into conical 96 well (Thermofisher) plate precoated with PBS 1% BSA and incubated overnight in Danilchik media (53 mM NaCl, 11.7 mM Na_2_CO_3_, 4.25 mM potassium gluconate, 2 mM MgSO_4_, 1 mM CaCl_2_, 17.5 mM Bicine, 1 mg/mL BSA, pH 8.3) until control embryos reached stage 20.

### Quantitative PCR

Quantitative real time PCR was done as previously described (Neuner et al., 2009). All primers were tested for efficiency. Embryos were collected at stage 15–18, B-16 cells were collected after 48 h of transfection and RNA was extracted using (Roche, RNA isolation Kit). Total RNA was quantified on a nanodrop (Thermofisher) at 260 nm. The cDNA was made using (Invitrogen Superscript III) according to manufacturer’s instructions. Quantitative PCR was performed using SYBR green (Takara, Kyoto, Japan) to measure mRNA levels of Adam11.L and GAPDH was used to normalize total cDNA amount, in case of B-16 cells beta-actin was used to normalize the total cDNA amount. The relative gene expression was calculated using the ΔΔCT method as described (Livak and Schmittgen, 2001). The result is represented in fold change compared to non-injected control. Oligos used are described in the table below (xl indicate *X. laevis* primer, all other primer are mouse).

**Table:** 

Target	Sequence 5’−3′
Adam11.L F xl	GGA​GCA​CTA​CAC​CCT​GAT​GG
Adam11.L R xl	CTG​GCT​TCT​AAC​CCT​GGT​GTC
GAPDH F xl	TTA​AGA​CTG​CAT​CAG​AGG​GCC​CAA
GAPDH R xl	GGG​CAA​TTC​CAG​CAT​CAG​CAT​CAA
E-cadherin F	CAT GCA GTT CTG CCA GAG GA
E-cadherin R	ATC AGA ATC AGC AGG GCG AG
N-cadherin F	TGT GGA GGC TTC TGG TGA AA
N-cadherin R	CTT GAA ATC TGC TGG CTC GC
vimentin F	AAC GAG TAC CGG AGA CAG GT
vimentin R	AGG TCA TCG TGA TGC TGA GAA
ß-actin F	GTC CAC CTT CCA GCA GAT GTG
ß-actin R	GCA TTT GCG GTG GAC GAT
CyclinD1 F	TGG TGA ACA AGC TCA AGT GG
CyclinD1 R	GCA GGA GAG GAA GTT GTT GG
Snail F	TCG GAA GCC TAA CTA CAG CGA
Snail R	AGA TGA GCA TTG GCA GCG AG
Adam11 F	GGT TAG TGC CTG TGA TGT GTA T
Adam11 R	GTG GGT ATG GAT TGA GGA CTT G

### Immunoprecipitation and western blots

Hek293T, embryos, Animal caps and CNC explants were extracted in 1XMBS-1% Triton-X100, Protease phosphatase inhibitor cocktail 1X (Thermoscientific) and 5 mM EDTA. Immunoprecipitations were performed using 1-2 ug of antibody bound to protein A/G magnetic beads (Thermofisher), incubated overnight at 4°C. The beads were washed 3 times for 5 min with extraction buffer at room temperature then eluted in 2X reducing laemmli sample buffer. All proteins were separated in 5%–22% gradient SDS-PAGE gels and transferred to polyvinylidene fluoride membranes (PVDF, Millipore, Billerica, MA) using a semi-dry transfer apparatus (Hoeffer). Quantitative capillary western blots were performed using Biotechne automatic western machine (Wes) according to manufacturer’s instructions https://www.bio-techne.com/resources/videos/meet-wes-protein-characterization-problem-solver. The data obtained using WES is a graph corresponding to the light intensity from the chemiluminescence. This graph can be shown as a conventional gel image as shown in Figure 1B or as an intensity histogram as shown in Figure 1C.

### Whole mount *in situ* hybridization

Whole mount *in situ* hybridization was done using previously described protocol (Harland, 1991). The probes for *Sox2, Snai2/Slug, Sox9, Sox8* were synthesized using diogoxigenin-rUTP–label.

### Immunofluorescence

CNC cells were dissected at stage 17 and placed on fibronectin coated glass bottom plates (20ug/mL) for 1–3 h at 18°C (Cousin and Alfandari, 2018). The explants were fixed using MEMFA (0.1 M MOPS pH 7.4, 2 mM EGTA pH 8, 1 mM MgSO4 and 4% paraformaldehyde) for 1 h, permeabilized using 0.5% TX100 in 1XMBS with 100 mM glycine for 1 h. The explants were then blocked using PBS containing 10% heat inactivated goat serum, 1% BSA, 0.1% Tween for 1 h prior to incubation in the same buffer with the primary antibodies overnight at 4°C. CNC explants were washed in PTw (PBS-tween 0.1%) 3 times 15 min, blocked again for 1 h at room temperature using blocking solution prior to incubation with the secondary antibody and Hoest33342 for 1 h at room temperature. The explants were washed 3 times in PTw and imaged using Nikon confocal microscope (A1RHD25).

### Immunofluorescence analysis

The Immunofluorescence images were analyzed using Fiji (Schindelin et al., 2012) the mean gray value was calculated for the antibody specific channel and was normalized using the mean gray value for DAPI. The images were taken at same gain and power for all explants within the same experiment. To account for the differences in-between biological repeats, in each repeat the NI was set to 1 and the values for MO11 were calculated relative to it. Edu + cells were counted manually using Fiji and normalized to the relative size of the explant.

### Mass spectrometry

Protein extracted from 10 CNC explants as described above or immunoprecipitated with the Flag antibody from embryos or Hek293T cells were diluted in 8M urea and processed with Trypsin/Lysine-C according to manufacturer instruction (Promega). Tandem mass spectrometry was performed using a Thermo orbitrap Fusion (Mass spectral data were obtained at the University of Massachusetts Mass Spectrometry Core Facility, RRID:SCR_019063). All MS/MS samples were analyzed using Sequest (Thermo Fisher Scientific, San Jose, CA, United States; version IseNode in Proteome Discoverer 2.4.1.15). Sequest was set up to search XenbaseProteinXL2020.fasta (unknown version, 72,266 entries) or the human protein database uniprot-human containing the sequences for *Xenopus* Adam11 (126,358 entries) assuming the digestion enzyme trypsin. Sequest was searched with a fragment ion mass tolerance of 0.60 Da and a parent ion tolerance of 10.0 PPM. Carbamidomethyl of cysteine was specified in Sequest as a fixed modification. Oxidation of methionine, acetyl of the n-terminus and phospho of serine threonine and tyrosine were specified in Sequest as variable modifications.

Criteria for protein identification: Scaffold (version Scaffold_5.0.1, Proteome Software Inc., Portland, OR) was used to validate MS/MS based peptide and protein identifications. Peptide identifications were accepted if they could be established at greater than 95.0% probability by the Peptide Prophet algorithm (Keller et al., 2002) with Scaffold delta-mass correction. Protein identifications were accepted if they could be established at greater than 99.0% probability and contained at least 2 unique identified peptides. Protein probabilities were assigned by the Protein Prophet algorithm (Nesvizhskii et al., 2003). Proteins that contained similar peptides and could not be differentiated based on MS/MS analysis alone were grouped to satisfy the principles of parsimony. Proteins sharing significant peptide evidence were grouped into clusters.

### Flow cytometry

Flow cytometry was performed on B-16F10 cells that were transiently transfected with the various plasmids. Only the cells successfully transfected with FUCCI were fluorescent and analyzed. The cells were harvested 36–48 h post transfection using a cell scraper and resuspended in PBS 1% BSA. Flow cytometry was performed on a BD DUAL LSRFortessa and the data was analyzed using FACSDiva 8.0. 50,000 cells were acquired for each sample in three independent experiments.

### Statistical analysis

If not specified in the figure legends, for two samples two-tail unpaired Student’s t-test was performed. For more than 2 samples one-way ANOVA was performed.

## Data Availability

The data presented in the study are deposited in the Harvard dataverse at: https://dataverse.harvard.edu/dataset.xhtml?persistentId=doi:10.7910/DVN/0MFJGM.

## Supplementary material

SUPPLEMENTARY FIGURE S1
**(A)** Volcano plot representing the fold change of protein expression in control CNC explants compared to CNC KD for Adam11 (*X*-axis). Fisher’s exact Test was applied; green squares represent proteins that are significantly up or downregulated. Green triangles represent proteins that are absent in one of the samples. **(B)** Venn diagram of proteins (minimum 1 high confidence peptide) statistically downregulated (41) or upregulated (81) in CNC explants from Adam11 KD (MO11) compared to non-injected (NI) control.

SUPPLEMENTARY FIGURE S2
**Heat Shock protein expression in CNC**. Lc/Ms/Ms of total protein extract from *Xenopus laevis* CNC. Proteins shown have 1 minimum high confidence peptide and have HSP in the description or name of the protein. The upper panel shows the statistical analysis of Non-injected CNC (NI) compared to CNC KD for Adam11 (MO11). The lower panel shows the statistical analysis of non-injected CNC to CNC KD for Adam13/33 (MO13). The quantitative profile indicates the proteins that are statistically differentially expressed in the samples. The first arrow is for the control (NI), and the second is for the experimental (MO11 or MO13). The number of exclusive unique peptides is indicated under each sample.

SUPPLEMENTARY FIGURE S3
**ADAM11 protein sequence alignment**. Single amino acid sequence alignment of *Xenopus* (upper line), Mouse (Middle line), and Human (Lower line). The red residues are identical, and the blue are similar. The start of each domain is indicated by a green arrowhead on top of the sequence (Pro; Pro domain, Meta; Metalloprotease, Dis; Disintegrin, CR; Cysteine Rich, TM; transmembrane, Cyto; Cytoplasmic tail). The protease “active” site is indicated by a green arrowhead. The transmembrane domain is underlined in green.

SUPPLEMENTARY MOVIE S1
**Time lapse during neurulation**. Embryos KD for Adam11 are in the top row (MO11), the non-injected (NI) controls are in bottom row. All embryos finished gastrulation at the same time.

SUPPLEMENTARY MOVIE S1
**Time lapse of grafted CNC migration**. Left is the control, right is KD for Adam11.
