# Supplementary figures and images for: ADAM11 a novel regulator of Wnt and BMP4 signaling in neural crest and cancer
Source: Front Cell Dev Biol. 2023 Sep 12;11:1271178. doi: 10.3389/fcell.2023.1271178 (PMC10520719; doi:10.3389/fcell.2023.1271178)

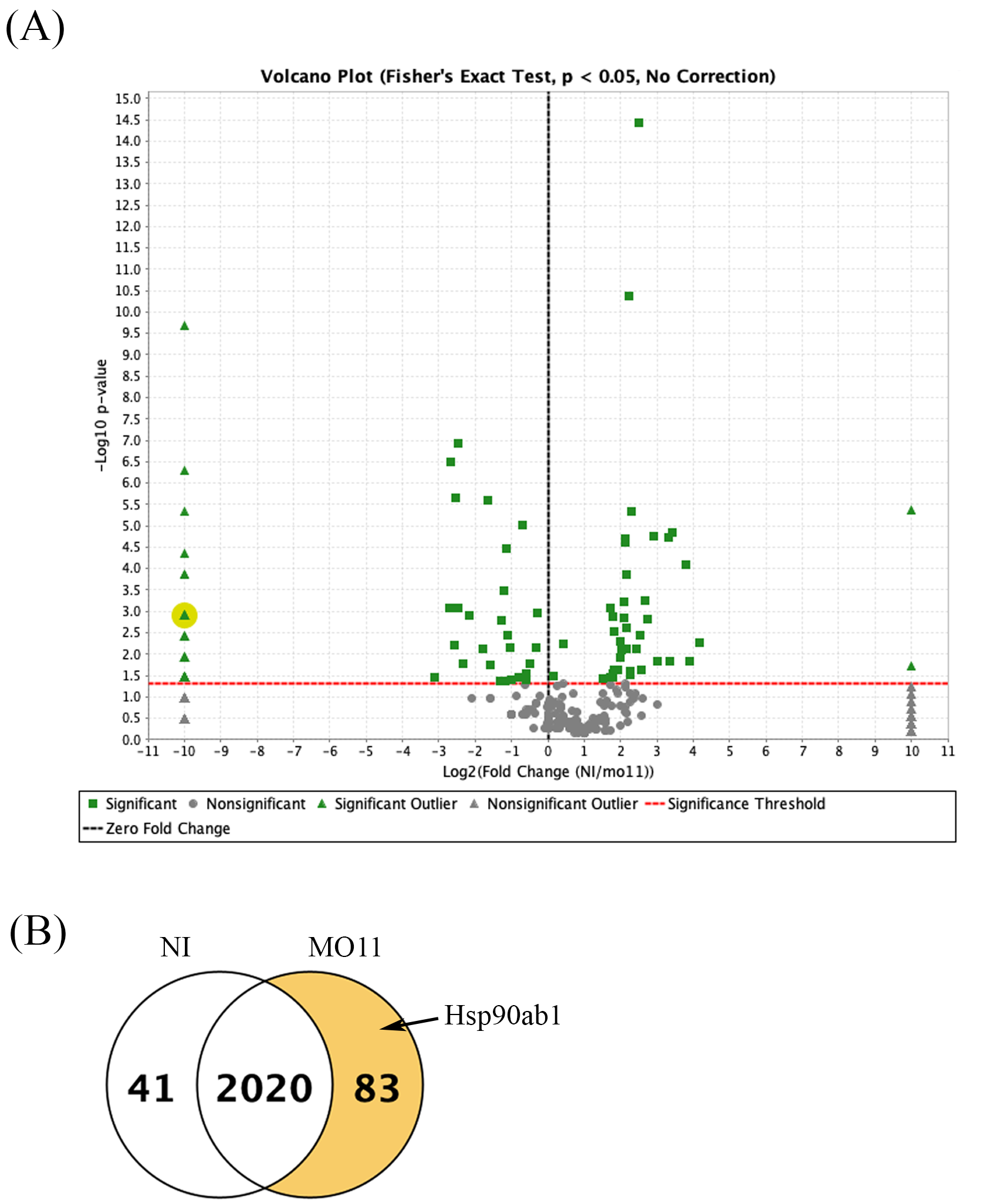

Supplement: Supplementary file 1 [file Presentation1.zip › Supplemental material/Fig.S1.tif]

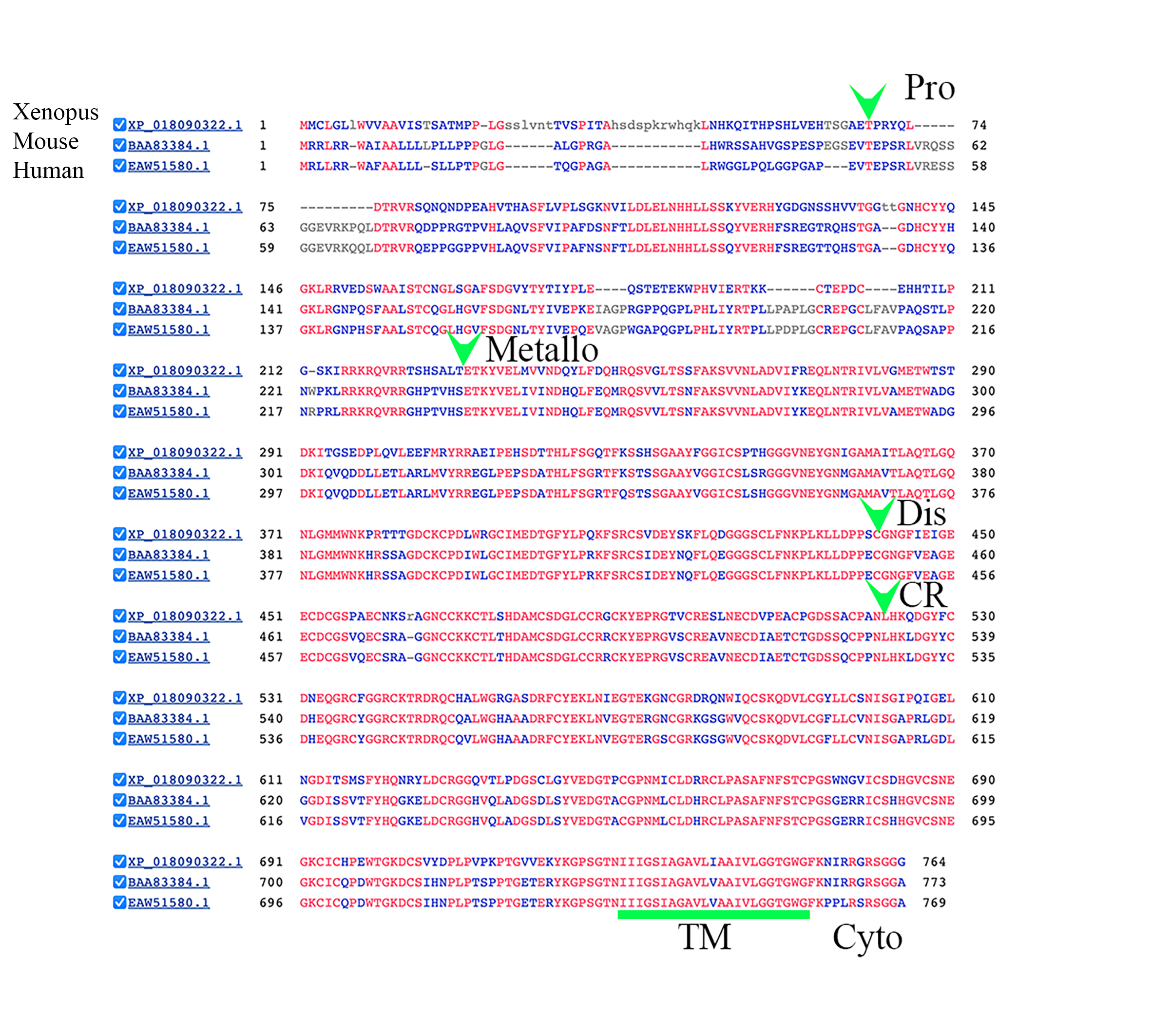

Supplement: Supplementary file 1 [file Presentation1.zip › Supplemental material/Fig.S3.tif]

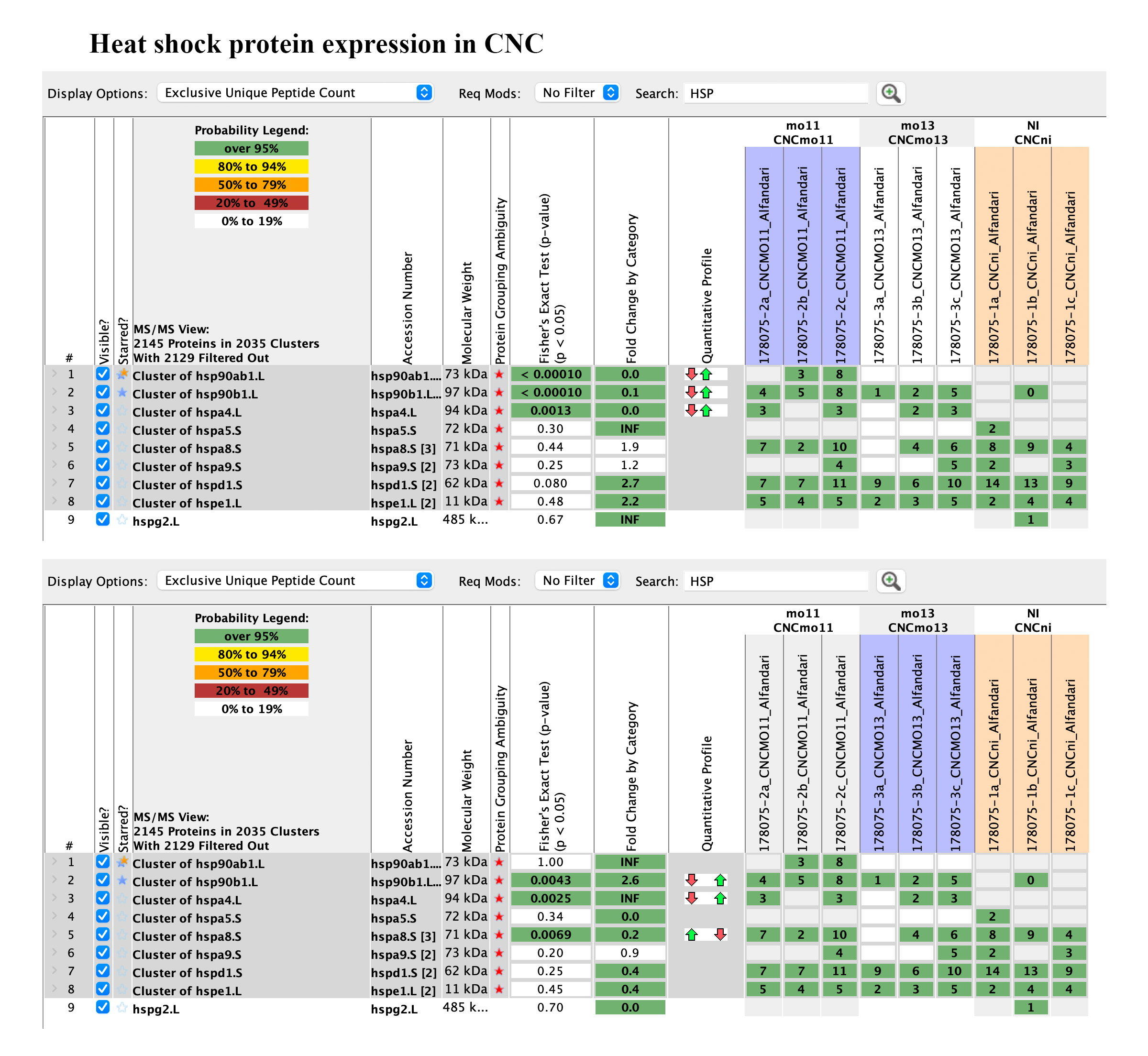

Supplement: Supplementary file 1 [file Presentation1.zip › Supplemental material/Fig.S2.tif]
